# Prevalence and associated factors of suicidal ideation, suicide plans, and suicide attempts among nurses: a systematic review and meta-analysis

**DOI:** 10.1186/s12888-026-08426-x

**Published:** 2026-07-30

**Authors:** Wen Huang, Bin Wan, Chuanrui Yang, Jiezheng Hu, Qilei Jia

**Affiliations:** 1 Department of Nursing, Suining Central Hospital, Suining, China; 2 School of Basic Medical Sciences & School of Nursing, Chengdu University, Chengdu, China; 3 Department of Nursing, Affiliated Hospital of Chengdu University, Chengdu, Sichuan China; 4 Department of Urology, Qingbaijiang District People’s Hospital of Chengdu, Chengdu, Sichuan China

**Keywords:** Nurses, Suicidal ideation, Suicide plans, Suicide attempts, Prevalence, Associated factors, Meta-analysis

## Abstract

**Background:**

Suicide-related outcomes among nurses represent an important occupational mental health concern; however, evidence regarding their prevalence and associated factors remains fragmented. This systematic review and meta-analysis aimed to estimate the pooled prevalence of suicidal ideation (SI) and suicide plans (SP) among nurses, narratively summarize suicide attempts (SA), and synthesize associated factors.

**Methods:**

A systematic search was conducted in PubMed, Scopus, Embase, PsycINFO, Web of Science, the Cochrane Library, and four Chinese databases (CNKI, CBM, VIP, and Wanfang) from inception to November 1, 2025. Random-effects models were used to estimate pooled prevalence, 95% confidence intervals (CIs), and 95% prediction intervals (PIs) for SI and SP. Subgroup analyses and meta-regression were conducted to explore heterogeneity. Publication bias for SI was assessed using a funnel plot and Egger’s test. Associated factors were synthesized narratively, and SA-related findings were summarized narratively because of limited available data and substantial heterogeneity.

**Results:**

A total of 1,840 records were initially identified, and 55 articles representing 54 independent studies across 18 countries were included. The pooled prevalence was 12.8% (95% CI: 10.1%–15.7%; 95% PI: 3.2%–36.3%; I² = 99.0%) for SI and 5.0% (95% CI: 3.8%–6.4%; 95% PI: 1.6%–17.6%; I² = 70.2%) for SP, indicating substantial heterogeneity for SI and moderate-to-high heterogeneity for SP. In subgroup analyses, SI prevalence varied by country, geographical region, and recall period. By country, SI prevalence ranged from 6.1% in Spain to 15.5% in Ireland. Regionally, Asia showed the highest SI prevalence (15.7%), followed by Europe (10.8%) and North America (8.6%). Across recall periods, SI prevalence was highest for past-week recall (18.1%) and lowest for past-month recall (8.1%). Meta-regression indicated a negative association between mean age and SI prevalence (R² = 25.86%). SA was reported in a limited number of studies and was summarized narratively because of heterogeneity in measurement and populations. Associated factors spanned multiple domains, with psychological and occupational factors most frequently reported.

**Conclusions:**

SI and SP are relatively common among nurses, whereas evidence on SA remains limited. The wide prediction intervals suggest that pooled prevalence estimates should be interpreted as summary measures of heterogeneous evidence rather than precise estimates applicable to all nurse populations. Suicide-related outcomes among nurses appear to be influenced by individual psychological factors as well as occupational and contextual conditions, highlighting the need for targeted mental health support and workplace-based preventive strategies.

**Supplementary Information:**

The online version contains supplementary material available at https://doi.org/10.1186/s12888-026-08426-x.

## Introduction

Suicide remains a major global public health concern and an important cause of preventable mortality. In 2021, an estimated 727,000 people died by suicide worldwide, and suicide was the third leading cause of death among individuals aged 15–29 years [1]. Suicide-related outcomes are commonly understood as a continuum, including suicidal ideation (SI), suicide plans (SP), and suicide attempts (SA), each representing a different level of suicide risk and requiring timely identification, assessment, and intervention [2, 3]. Although suicide and suicidal behaviours have been widely studied in the general population and among individuals with mental disorders, increasing attention has been directed toward occupational groups exposed to sustained psychological stress, emotional burden, and adverse working conditions [4]. Among these occupational groups, nurses deserve particular attention because of the size of the nursing workforce and the demanding nature of nursing work.

Nurses constitute the largest occupational group in the health workforce and play a critical role in maintaining healthcare systems worldwide. Globally, there are approximately 29 million nurses, yet the World Health Organization estimates a shortage of 4.5 million nurses by 2030 [5]. In clinical care settings, nurses are often exposed to high work demands, substantial emotional burden, and considerable responsibility-related pressure. They frequently face occupational challenges such as shift work, inadequate staffing, exposure to patient suffering and death, workplace violence, and moral distress [6–10]. Existing evidence suggests that nurses may face a higher risk of suicide-related outcomes than the general population or some other occupational groups [11–13]. Therefore, suicide-related outcomes among nurses are not only individual mental health concerns, but also issues related to the stability of healthcare systems and the quality of patient care.

Previous studies have examined suicide-related outcomes among nurses in different countries and clinical settings, suggesting that SI, SP, and SA may all represent important mental health concerns within the nursing workforce. Some studies have reported that the proportion of nurses experiencing suicidal ideation can exceed one quarter, and suicide plans and suicide attempts have also been observed in certain samples [14–17]. Meanwhile, previous studies have reported a range of factors associated with suicide-related outcomes among nurses, including demographic characteristics, mental health status, occupational stress, work environment, and social support [16, 18–20]. However, findings regarding these associated factors have not been entirely consistent across studies. Therefore, in addition to estimating the prevalence of suicide-related outcomes among nurses, it is also necessary to further synthesize the factors reported to be associated with these outcomes.

Previous reviews have examined suicide-related outcomes from the perspectives of healthcare workers, nursing personnel, or nurses, providing an important foundation for this field [21–24]. However, existing reviews have mainly focused on broad mapping of suicide-related evidence or included wider healthcare and nursing personnel categories. For practicing nurses with recognized professional qualifications working in healthcare settings, quantitative synthesis of the pooled prevalence of SI and SP, together with evidence on factors associated with SI, SP, and SA, remains limited. Therefore, it is necessary to further focus on practicing nurses, distinguish between SI, SP, and SA as distinct suicide-related outcomes, and conduct a systematic review and meta-analysis of prevalence evidence and associated factors. Such evidence synthesis may help clarify the mental health burden among practicing nurses and provide more targeted evidence for occupational mental health screening, workplace support policies, and suicide prevention interventions.

Therefore, this systematic review and meta-analysis aimed to estimate the pooled prevalence of SI and SP among practicing nurses and to narratively summarize the prevalence of SA when nurse-specific data were available. In addition, this review aimed to synthesize factors associated with SI, SP, and SA among practicing nurses.

## Methods

This systematic review and meta-analysis was pre-registered with PROSPERO (CRD420251186407) and conducted in accordance with the Preferred Reporting Items for Systematic Reviews and Meta-Analyses (PRISMA) 2020 guidelines [25]. One amendment to the registered protocol was that SA prevalence was summarized narratively rather than meta-analysed because of limited and heterogeneous nurse-specific data.

### Search strategy

Two independent researchers (Wen Huang and Bin Wan) performed systematic literature searches across six international bibliographic databases, including PubMed, Scopus, Embase, PsycINFO, the Cochrane Library, and Web of Science, and four Chinese bibliographic databases, including China National Knowledge Infrastructure (CNKI), Chinese Biomedical Literature Database (CBM), VIP Chinese Science and Technology Journal Database (VIP), and Wanfang Data, from database inception to November 1, 2025. Chinese professional terminology corresponding to the English search terms was used for searches in Chinese databases to ensure consistency across languages.

The search strategy was developed around three core concepts: the target population, SI, SP, and SA, and epidemiological or association-related terms. The population search block included controlled vocabulary and free-text terms related to nurses and nursing personnel. The SI, SP, and SA search block included terms related to suicidal ideation, suicide planning, suicide attempt, suicidality, and other relevant suicide-related terms. The third block included broad epidemiological and association-related terms, such as prevalence, epidemiology, frequency, correlates, associated factors, risk factors, predictors, and determinants, to reflect the dual focus of this review on prevalence estimates and factors associated with SI, SP, and SA among nurses.

Controlled vocabulary terms, including Medical Subject Headings (MeSH), Emtree terms, and database-specific subject headings, were combined with free-text keywords and adapted to the syntax of each database. Boolean operators (AND/OR) were used to combine search terms within and across the three concept blocks. In addition, the reference lists of eligible articles were manually screened to identify additional relevant records. The detailed search strategy for each database is provided in Supplementary Table S1.

### Selection criteria

Two reviewers (Wen Huang and Bin Wan) independently screened the titles, abstracts, and full texts of all retrieved records. Disagreements were resolved through discussion, and any unresolved discrepancies were adjudicated by a third reviewer (Chuanrui Yang).

Studies were eligible for inclusion if they met the following criteria: (1) the participants were adult registered nurses, licensed nurses, or otherwise qualified practicing nurses working in healthcare settings, including hospitals, clinics, nursing homes, or other clinical care settings; (2) the article reported the prevalence of SI, SP, or SA, provided sufficient data for prevalence calculation, or reported factors associated with SI, SP, or SA among eligible nurses; (3) when nurses were included as a subgroup within a broader healthcare worker sample, nurse-specific data on prevalence or associated factors were available; and (4) the full-text article was published in English or Chinese. According to the type of data reported, articles were included in the prevalence meta-analysis, the narrative synthesis of associated factors, or both.

Studies were excluded if they met any of the following criteria: (1) they did not report the prevalence of SI, SP, or SA, did not provide extractable prevalence data, and did not report factors associated with SI, SP, or SA among eligible nurses; (2) the study population was labelled as “nurses” but included non-eligible nursing-related personnel, such as nursing students, nursing assistants, nurse aides, or caregivers, and data for eligible nurses could not be separated; (3) the full text was unavailable despite reasonable attempts to obtain it, including contacting corresponding authors when necessary; (4) the article type was not eligible, including intervention studies or programme evaluations without extractable nurse-specific baseline or cross-sectional data, editorials, case reports, case series, meeting abstracts, commentaries, theses, and dissertations; (5) the nurse subgroup included fewer than 50 participants; or (6) the study was rated as having low methodological quality based on the corresponding critical appraisal tool. The minimum sample size criterion was applied to reduce the influence of unstable estimates derived from very small samples, particularly for relatively uncommon outcomes such as SP and SA. Studies classified as having low methodological quality were excluded from the final review and evidence synthesis.

For the narrative synthesis of associated factors, articles were eligible when they formally examined factors in relation to SI, SP, and/or SA among eligible nurses, regardless of whether the reported associations were statistically significant.

### Data extraction

Two independent researchers (Wen Huang and Bin Wan) extracted data using a pre-designed standardized form. For the characteristics of included articles, the extracted information included first author, publication year, country or region, survey year, pandemic context, study design, sampling method, study setting or participant group, sample size, mean age, female proportion, and outcomes reported.

For prevalence-related outcomes, we extracted outcome type, recall period, measurement instrument or item, overall event number and total sample size, overall prevalence, and sex-specific event numbers and prevalence estimates where available. We also recorded whether each estimate was eligible for inclusion in the outcome-specific meta-analysis. When SI, SP, or SA was assessed but extractable event counts or prevalence estimates were not available, the outcome was retained for descriptive reporting but was not included in the quantitative prevalence synthesis. “NR” was used when information was not reported, and “NA” was used when a field was not applicable.

Associated factors of SI, SP, and SA were extracted for narrative synthesis rather than quantitative pooling because of heterogeneity in definitions, statistical models, covariate adjustment, and effect measures across studies. For study-level extraction, only factors that were empirically examined in relation to SI, SP, or SA among nurses were extracted. Factors were classified as significant adjusted associations when they were statistically significant in multivariable or otherwise adjusted models; as significant unadjusted associations when they were statistically significant in crude, bivariate, or univariable analyses but no significant adjusted association was available; and as reported non-significant associations when they were examined but not statistically significant in the original studies. When adjusted estimates were available, they were prioritized for interpretation. Extracted factors were grouped into six domains: sociodemographic and socioeconomic; psychological and psychiatric; physical health, sleep, and lifestyle; occupational; social and organizational; and early-life adversity and pandemic-related contextual factors. Findings were interpreted as observational associations rather than causal risk factors, given the predominance of cross-sectional study designs.

When an article reported multiple estimates for the same outcome, all relevant estimates were extracted, and the estimate used in the quantitative synthesis was selected according to the predefined data synthesis rules. When multiple publications were known or suspected to be based on the same original dataset or overlapping samples, relevant information was extracted from each article, and potentially overlapping estimates were flagged for handling during quantitative synthesis. Corresponding authors were contacted to retrieve missing or unclear data when necessary and feasible.

### Quality assessment

Two researchers (Wen Huang and Bin Wan) independently assessed the methodological quality of eligible studies using design-specific appraisal tools. The Joanna Briggs Institute (JBI) critical appraisal checklist was applied to cross-sectional studies [26], and the Newcastle–Ottawa Scale (NOS) was used for the longitudinal study [27]. For cross-sectional studies, each item was rated as “Yes”, “No”, “Unclear”, or “Not applicable”. Overall quality scores were calculated based on the number of “Yes” responses across eight items, with scores of 7–8 classified as high quality, 4–6 as moderate quality, and 0–3 as low quality. Studies classified as having low methodological quality were excluded from the final review and evidence synthesis. For the longitudinal study, an NOS score ≥ 6 indicated acceptable methodological quality, whereas a score < 6 was considered methodologically inadequate and would result in exclusion. When multiple publications were derived from the same dataset, methodological quality was assessed once at the study level to avoid duplication. Discrepancies between reviewers were resolved through discussion, and a third reviewer (Chuanrui Yang) was consulted when necessary.

### Data synthesis and statistical analyses

All statistical analyses were performed using Stata 18.0. The primary outcomes were pooled prevalence estimates of SI and SP with corresponding 95% confidence intervals (CIs). For each study, the number of events and total sample size were entered directly into Stata. Prevalence estimates were pooled using DerSimonian–Laird random-effects models with the Freeman–Tukey double-arcsine transformation and back-transformed for presentation. To facilitate interpretation of between-study heterogeneity, 95% prediction intervals (PIs) were additionally calculated for SI and SP using random-effects meta-analysis with restricted maximum likelihood estimation on the logit-transformed prevalence scale and then back-transformed to the original proportion scale. Exact CIs were used for study-specific estimates. SA was not meta-analysed and was instead summarized narratively due to limited eligible studies and substantial clinical and methodological heterogeneity.

Only studies based on general or non-selected clinical nurse samples were included in the quantitative synthesis. Studies involving selected, event-exposed, help-seeking, service-based, or high-risk populations were excluded from pooled analyses but retained for narrative synthesis when nurse-specific data were available. To avoid double counting, multiple publications derived from the same dataset or overlapping samples were not simultaneously included. When multiple estimates were reported within a study, only one estimate per outcome was selected based on relevance and comparability.

Statistical heterogeneity was assessed using the I² statistic, with values > 75% indicating substantial heterogeneity. Subgroup analyses were conducted for SI where sufficient data were available, including study location, geographical region, World Bank income level of the study location, demographic, methodological, and contextual factors. Study locations were classified according to the World Bank income classification for countries and economies at the time of analysis; for studies conducted in Hong Kong or Taiwan, the corresponding economy-level income classification was used. Each subgroup required at least three studies per category. Due to limited data, subgroup analyses and meta-regression were not performed for SP.

Meta-regression analyses were conducted for SI to explore potential sources of heterogeneity, including survey year, log-transformed sample size, female proportion (per 10% increase), and mean age (per 10-year increase). Regression coefficients were estimated on the Freeman–Tukey double-arcsine transformed prevalence scale using random-effects meta-regression with restricted maximum likelihood estimation. Results were interpreted at the study level and not as causal or individual-level associations.

Sensitivity analyses were performed by sequentially excluding one study at a time. Publication bias was assessed using funnel plots and Egger’s test when at least 10 studies were available; otherwise, tests were not conducted due to limited power. A *p* value < 0.05 was considered statistically significant.

Associated factors of SI, SP, and SA were synthesized narratively and were not quantitatively pooled because of heterogeneity in definitions, statistical models, covariate adjustment, and effect measures across studies. The narrative synthesis summarized factors across six predefined domains: sociodemographic and socioeconomic; psychological and psychiatric; physical health, sleep, and lifestyle; occupational; social and organizational; and early-life adversity and pandemic-related contextual factors. Findings were interpreted as observational associations rather than causal risk factors.

## Results

### Study selection

**Fig. 1:**
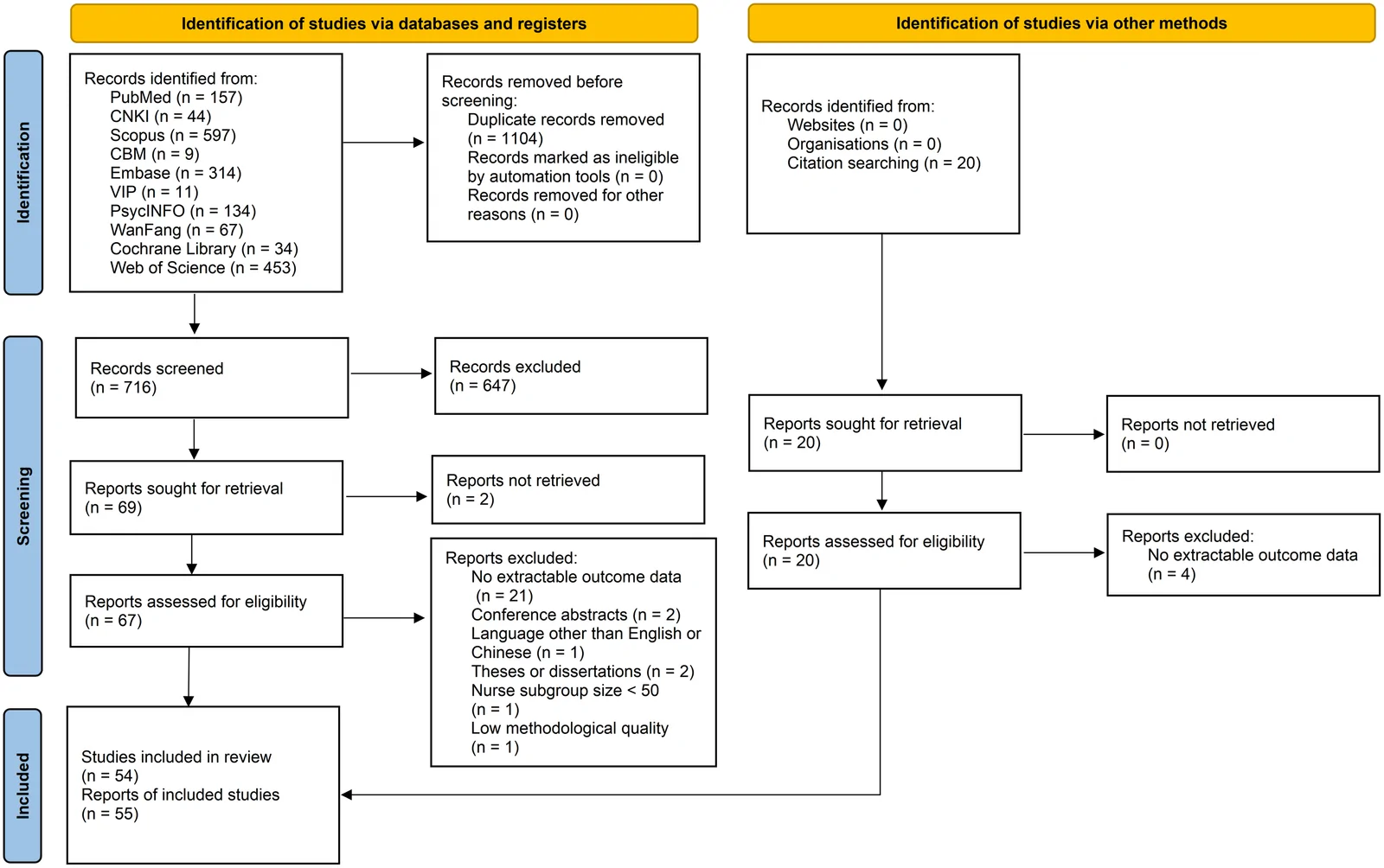
PRISMA flow diagram

A total of 1,840 records were identified through the literature search, including 1,820 records retrieved from electronic databases and 20 additional records identified through citation searching. After removal of 1,104 duplicate records from the database search results, 716 database records remained for title and abstract screening. Subsequently, 69 full-text articles retrieved from database searches were sought for retrieval, of which 2 could not be obtained. Therefore, 67 full-text articles were assessed for eligibility, and 28 articles were excluded for the reasons presented in Fig. 1. In addition, 20 articles identified through citation searching were successfully retrieved and assessed for eligibility, among which 4 articles were excluded. Ultimately, 55 articles met the eligibility criteria and were included in this review. As two articles reported data from the same study population, the final sample represented 54 independent studies. The study selection process is illustrated in Fig. 1.

### Study characteristics

A total of 55 articles [13–20, 28–74] were included in the review, representing 54 independent studies due to duplicate reporting of one dataset across publications. Detailed characteristics of the included studies are presented in Supplementary Table S2a. The handling of confirmed or potentially overlapping samples is shown in Supplementary Table S2b, and extracted prevalence-related data for SI, SP, and SA, including inclusion in the meta-analysis, are provided in Supplementary Table S3.

The studies were published between 2012 and 2025 and included nurse populations from 18 countries. China was the most frequently represented setting, followed by the United States, Ireland, Spain, and other countries across Asia, Europe, North America, South America, Africa, and Oceania. Reported survey periods ranged from 2008 to 2024, although several studies did not clearly report the data collection period. Most studies used a cross-sectional design, with only one longitudinal study identified [52]. Non-random sampling was more common than random sampling, and a substantial proportion of studies were conducted during the COVID-19 pandemic.

The studies covered a wide range of clinical settings and nursing specialties, including registered or licensed nurses, hospital nurses, psychiatric nurses, operating room nurses, oncology nurses, nursing home nurses, intensive care unit (ICU) nurses, and nurse subgroups within broader healthcare worker samples. Three studies included selected, event-exposed, or risk-enriched populations, including nurses involved in patient safety incidents [60], nurse subgroups derived from a healthcare worker suicide-risk screening programme [70], and nurses identified as having moderate-to-high-risk adverse mental health outcomes, including suicidal ideation [71]. These studies were retained for narrative synthesis when relevant but were excluded from pooled prevalence analyses of general or non-selected nurse populations.

SI was the most frequently assessed outcome. Overall, 52 articles [13–20, 28–45, 48–50, 52–74] assessed SI, representing 51 independent studies due to duplicate reporting of one dataset; 8 studies [14, 15, 18, 37, 43, 47, 56, 70] assessed SP; and 8 studies assessed SA [14, 16, 18, 46, 47, 51, 59, 70]. Among these, 46 studies provided extractable SI prevalence data, 8 provided SP prevalence data, and 8 provided SA prevalence data. After applying eligibility criteria for quantitative synthesis, 39 studies [13–19, 29–43, 48, 49, 53–59, 61–68] were included in the SI prevalence meta-analysis and 7 studies [14, 15, 18, 37, 43, 47, 56] were included in the SP prevalence meta-analysis. SA prevalence was synthesized narratively rather than pooled quantitatively. In addition, 31 studies [13, 14, 16–20, 28, 33–37, 39–41, 44, 45, 49, 50, 52, 53, 58, 60, 62, 67, 69, 71–74] reported associated factors of SI, SP, and/or SA, representing 30 independent studies due to duplicate reporting of the same dataset across publications. These studies were included in the narrative synthesis of associated factors.

### Risk of bias

Methodological quality and risk of bias were assessed at the level of independent study units, with each unique dataset or non-overlapping sample counted once. When multiple publications were derived from the same dataset, such as Ding et al. [39] and Li et al. [72], they were consolidated and treated as a single study unit to avoid duplicate assessment. One study was excluded after methodological quality appraisal because it was rated as having low methodological quality. The final review therefore included 54 independent studies, comprising 53 cross-sectional studies and one longitudinal study. Among the 53 cross-sectional studies assessed using the Joanna Briggs Institute (JBI) critical appraisal checklist, 35 were rated as high quality and 18 as moderate quality; therefore, no low-quality cross-sectional studies remained in the final review. The single longitudinal study, assessed using the Newcastle–Ottawa Scale (NOS), scored 7/9, indicating acceptable methodological quality. The most common limitation was inadequate control of potential confounders. Detailed quality assessment results are presented in Supplementary Tables S4a and S4b.

### Prevalence of SI, SP, and SA

**Fig. 2:**
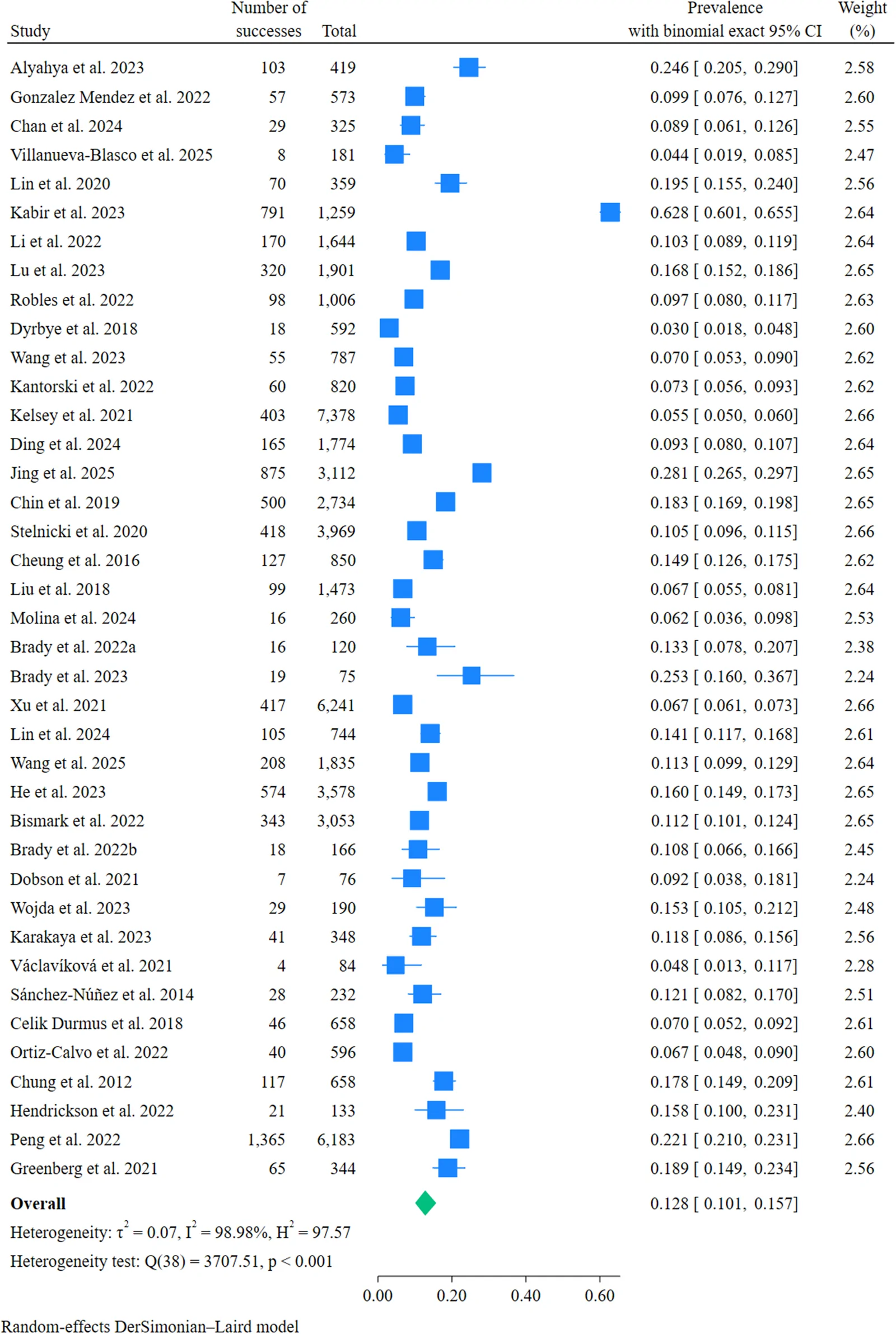
Forest plot of SI prevalence

**Fig. 3:**
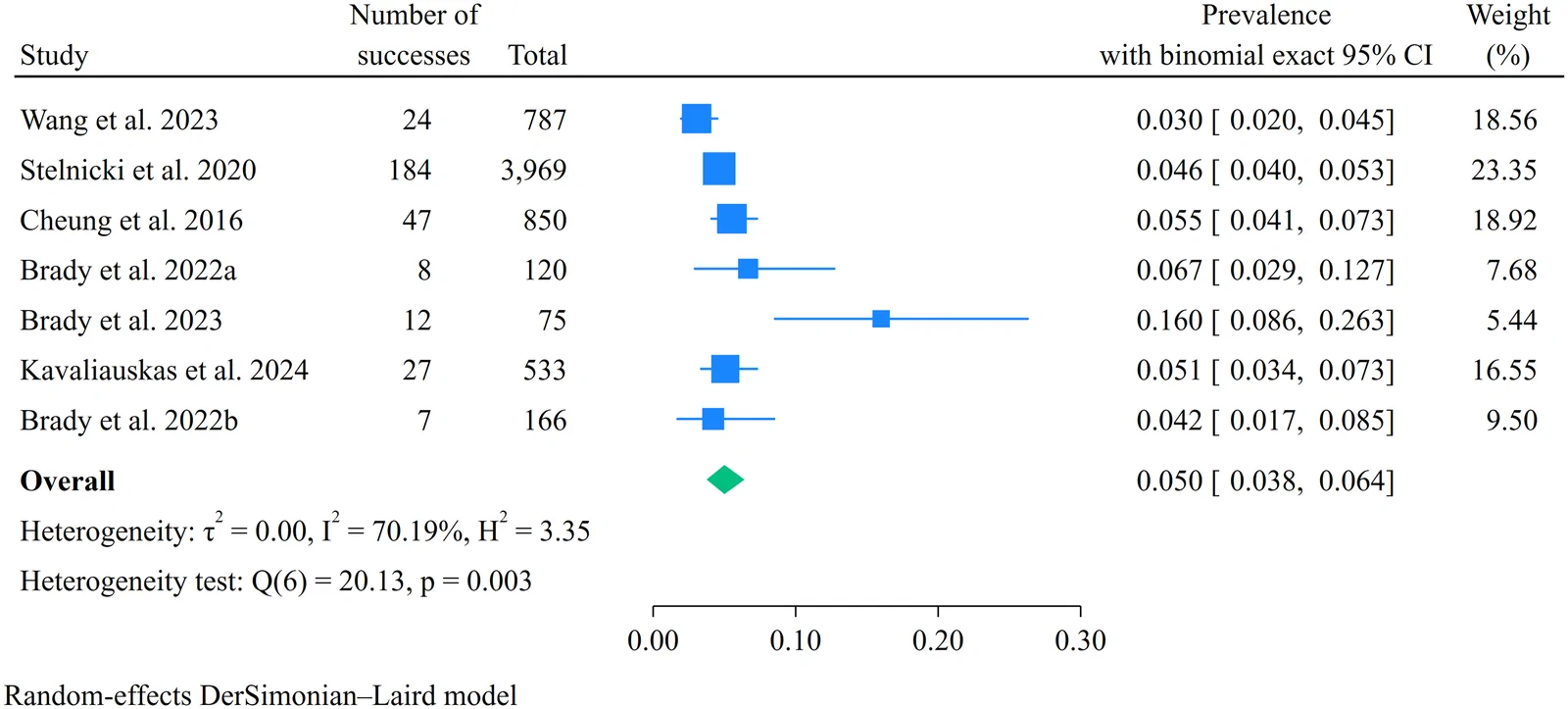
Forest plot of SP prevalence

After applying the criteria for quantitative synthesis, 39 studies [13–19, 29–43, 48, 49, 53–59, 61–68] were included in the SI meta-analysis and 7 studies [14, 15, 18, 37, 43, 47, 56] were included in the SP meta-analysis. The pooled prevalence of SI was 12.8% (95% CI: 10.1%–15.7%), with substantial heterogeneity (I² = 99.0%, *p* < 0.001). The corresponding 95% PI ranged from 3.2% to 36.3%, indicating considerable between-study variability. The pooled prevalence of SP was 5.0% (95% CI: 3.8%–6.4%), with moderate-to-high heterogeneity (I² = 70.2%, *p* = 0.003). The corresponding 95% PI ranged from 1.6% to 17.6%. Forest plots are shown in Figs. 2 and 3.

SA prevalence was synthesized narratively rather than quantitatively pooled due to limited nurse-specific estimates and substantial heterogeneity in recall periods, sample characteristics, and measurement approaches. Eight studies provided extractable SA data (Supplementary Table S3). Among general or clinical nurse samples, past-year SA prevalence ranged from 0.7% in Canadian nurses [14] to 10.8% among nurses in tertiary hospitals in China [16]. Lifetime/ever SA estimates ranged from 1.5% among licensed nurses in Lithuania [47] to 9.6% among nurses in a tertiary teaching hospital in Nigeria [51]. One study from Turkey reported SA history prevalence of 3.2%, although the recall period was not clearly specified [59]. In addition, Zhang et al. [70], based on a self-selected suicide-risk screening sample rather than a general nurse population, reported a two-week SA prevalence of 2.36% and a lifetime prevalence of 8.76%. Overall, SA prevalence estimates varied across studies, populations, and measurement contexts; therefore, results were interpreted descriptively rather than as directly comparable pooled estimates.

### Subgroup analysis

Subgroup analyses were conducted to explore sources of heterogeneity in SI prevalence, and the results are presented in Table 1. Significant differences were observed across countries, geographical regions, and recall periods. By country, pooled SI prevalence ranged from 6.1% in Spain to 15.5% in Ireland (*P* < 0.001). Asia showed the highest prevalence, followed by Europe and North America (*P* = 0.031). Recall period significantly influenced SI prevalence (*P* < 0.001), with the highest estimate for past-week SI (18.1%), followed by past-two-week (14.1%), past-year (9.0%), and past-month (8.1%). No significant subgroup differences were observed for measurement instrument, pandemic context, World Bank income level of the study location, sex, sampling method, or clinical sample type.

**Table 1:** Subgroup analysis of SI prevalence among nurses

Subgroup variable	Category (No. of studies)	Prevalence (%)	95% CI	I² (%)	*P* for heterogeneity	*P* for subgroup differences
Country	China (16)	13.8	10.5–17.5	98.9	< 0.001	< 0.001
	United States (4)	7.1	4.1–10.8	90.3	< 0.001	
	Ireland (3)	15.5	8.6–23.9	74.3	0.020	
	Spain (3)	6.1	4.7–7.7	0.0	0.560	
Geographical region	Asia (20)	15.7	11.4–20.5	99.3	< 0.001	0.031
	North America (7)	8.6	6.0–11.7	95.8	< 0.001	
	Europe (9)	10.8	7.0–15.3	88.0	< 0.001	
Recall period	Past week (5)	18.1	13.8–22.9	81.1	< 0.001	< 0.001
	Past 2 weeks (14)	14.1	10.2–18.5	98.8	< 0.001	
	Past month (5)	8.1	6.7–9.6	56.5	0.056	
	Past year (7)	9.0	6.0–12.6	98.1	< 0.001	
Measurement instrument/item	PHQ-9 item 9 (13)	12.6	9.2–16.4	98.4	< 0.001	0.903
	Single-item SI question (12)	10.9	7.1–15.4	99.0	< 0.001	
	Instrument-derived SI items (7)	10.8	7.0–15.3	95.8	< 0.001	
	C-SSRS-based item (5)	11.0	6.5–16.4	84.3	< 0.001	
Pandemic context	During COVID-19 (27)	13.6	10.0–17.7	99.1	< 0.001	0.293
	Pre-COVID-19 (10)	10.8	7.4–14.8	98.3	< 0.001	
World Bank income level of study location	Upper-middle income (18)	12.1	9.0–15.5	98.7	< 0.001	0.833
	High income (20)	11.5	8.9–14.3	96.9	< 0.001	
Sex	Female (11)	13.5	9.7–17.9	98.3	< 0.001	0.672
	Male (8)	14.4	9.2–20.4	78.6	< 0.001	
Sampling method	Non-random sampling (31)	13.2	10.0–16.8	99.0	< 0.001	0.485
	Random sampling (8)	11.1	6.7–16.5	98.7	< 0.001	
Clinical sample type	General nurses/general clinical samples (31)	12.9	9.8–16.4	99.2	< 0.001	0.287
	Specialty or unit-specific clinical settings (6)	10.3	7.0–14.0	92.0	< 0.001	

Note. World Bank income level refers to the World Bank income classification for countries and economies at the time of analysis. For studies conducted in Hong Kong or Taiwan, the corresponding economy-level income classification was used. CI, confidence interval; PHQ-9, Patient Health Questionnaire-9; C-SSRS, Columbia-Suicide Severity Rating Scale. I² and *P* for heterogeneity indicate within-subgroup heterogeneity for each subgroup category, whereas *P* for subgroup differences indicates between-subgroup differences across categories within each subgroup variable

### Meta-regression analysis

Meta-regression analyses were conducted to examine associations between selected study-level characteristics and SI prevalence among nurses. The results are presented in Table 2. Among the moderators examined, mean age was significantly associated with SI prevalence. Each 10-year increase in mean age was associated with lower SI prevalence estimates (coefficient = − 0.686, 95% CI: −1.225 to − 0.147; *P* = 0.013), accounting for 25.86% of the between-study heterogeneity. In contrast, survey year, log-transformed sample size, and female proportion (per 10% increase) were not significantly associated with SI prevalence. Residual heterogeneity remained high across all meta-regression models, with I² values ranging from 98.45% to 98.89%.

**Table 2:** Meta-regression analysis of SI prevalence among nurses

Moderator	No. of studies	Regression coefficient	95% CI	*P* value	*R*² (%)	Residual I² (%)
Survey year	37	0.024	−0.051 to 0.099	0.529	0.00	98.65
Sample size, log-transformed	39	0.033	−0.151 to 0.216	0.728	0.00	98.66
Female proportion, per 10% increase	28	−0.347	−0.753 to 0.060	0.095	7.26	98.45
Mean age, per 10-year increase	17	−0.686	−1.225 to − 0.147	0.013	25.86	98.89

Note. CI, confidence interval. Regression coefficients are presented on the Freeman–Tukey double-arcsine transformed prevalence scale. Random-effects meta-regression models were fitted using restricted maximum likelihood estimation. R² indicates the proportion of between-study variance explained by the moderator

### Sensitivity analysis and publication bias

**Fig. 4:**
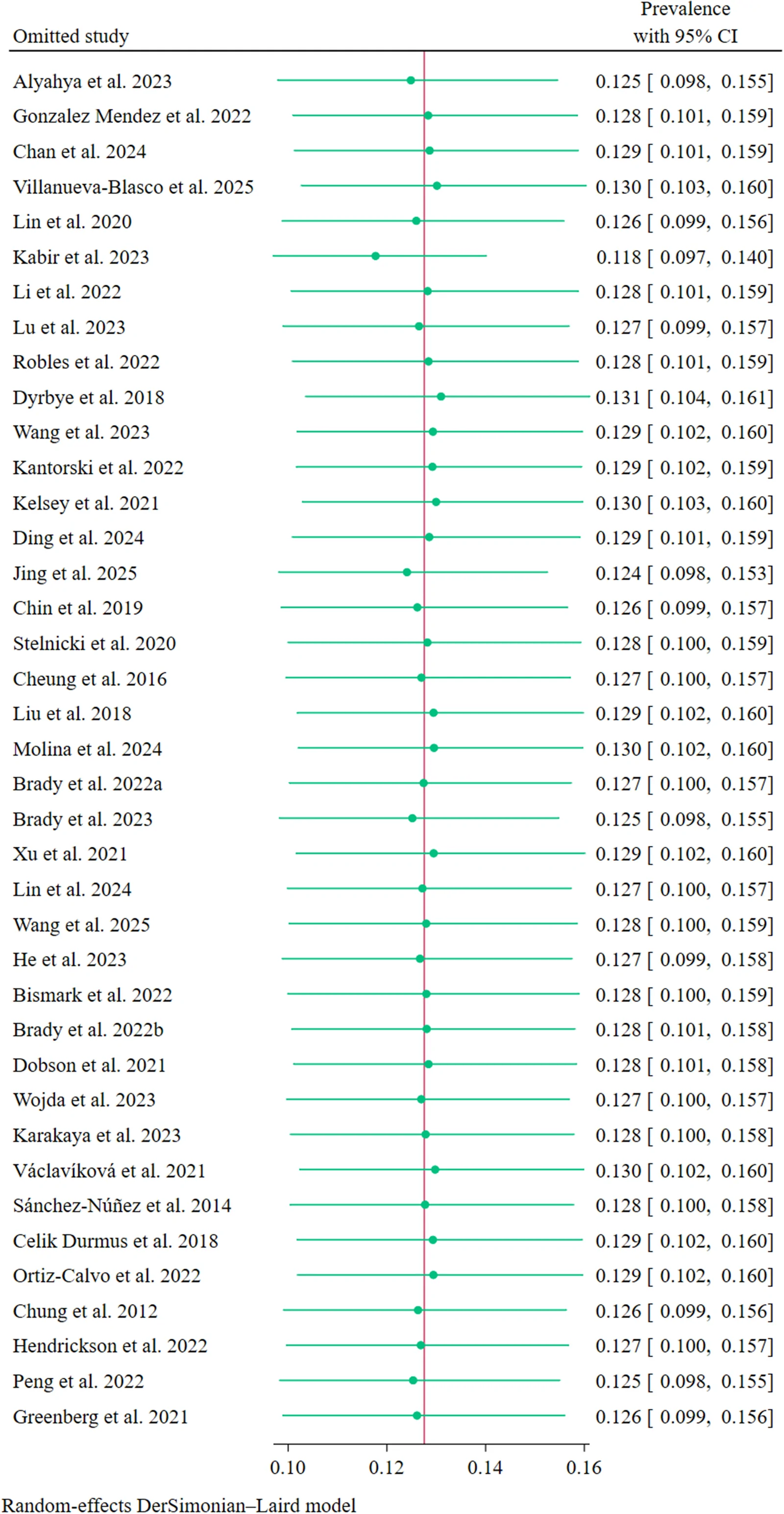
Leave-one-out sensitivity analysis for the pooled prevalence of SI

Leave-one-out sensitivity analyses were conducted for the pooled prevalence of SI and SP. For SI, sequential omission of individual studies did not materially change the pooled estimate (Fig. 4). Similar results were observed for SP (Supplementary Fig. S1).

**Fig. 5:**
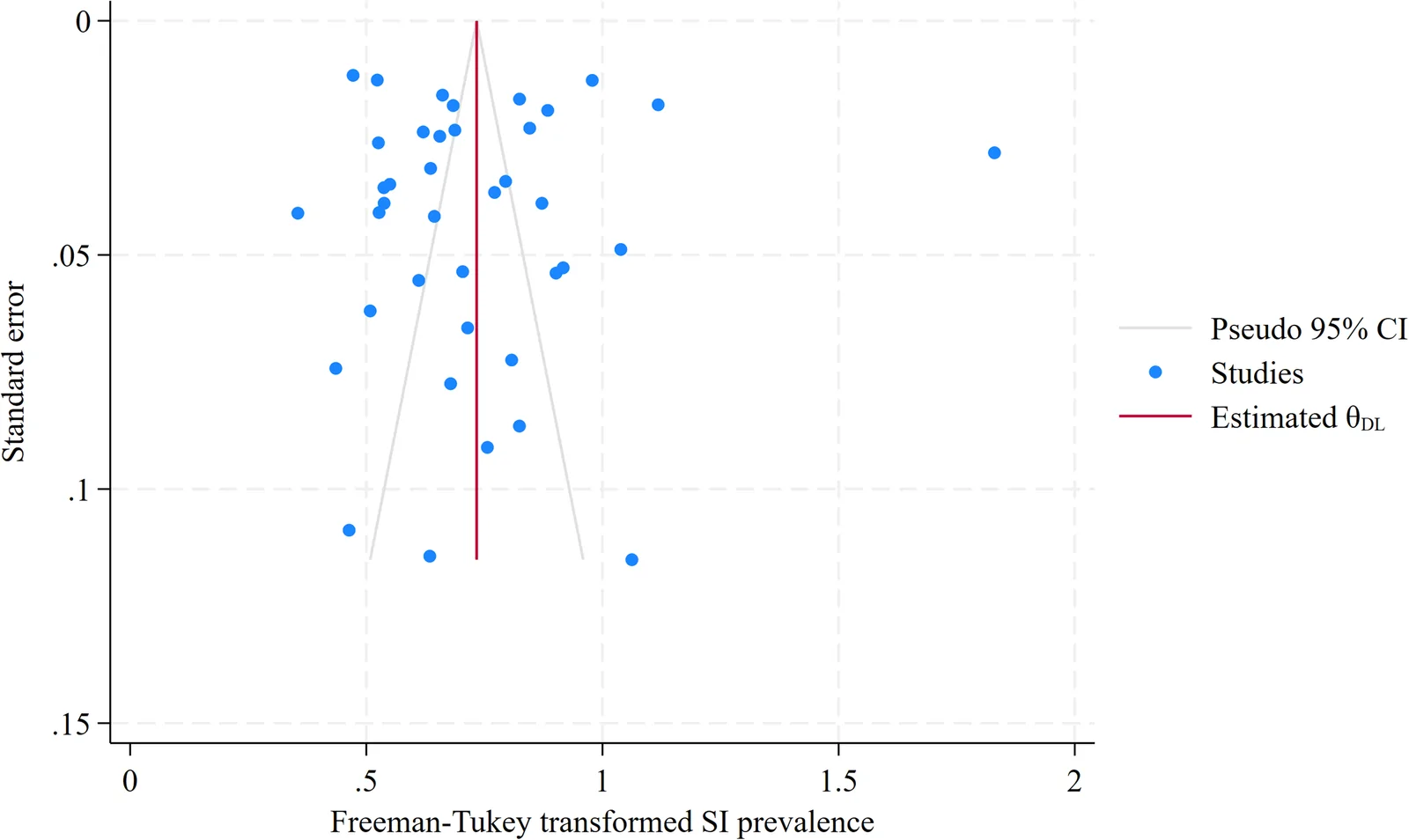
Funnel plot of suicidal ideation prevalence on the Freeman–Tukey double-arcsine transformed scale

Publication bias and small-study effects were assessed for SI using funnel plot inspection and Egger’s test, as at least ten studies were available. Visual inspection of the funnel plot did not provide clear evidence of asymmetry (Fig. 5), and Egger’s test indicated no significant small-study effects (*p* = 0.708). Publication bias was not assessed for SP due to the limited number of included studies (< 10).

### Associated factors of SI, SP, and SA

A total of 30 independent studies [13, 14, 16–20, 28, 33–37, 39–41, 44, 45, 49, 50, 52, 53, 58, 60, 62, 67, 69, 71–74] were included that examined factors associated with SI, SP, and/or SA among nurses. Detailed study-level findings, including both statistically significant and non-significant factors, are provided in Supplementary Table S5, and the domain-level summary is presented in Table 3. The extracted factors were grouped into six domains: sociodemographic and socioeconomic factors; psychological and psychiatric factors; physical health, sleep, and lifestyle-related factors; occupational factors; social and organizational factors; and early-life adversity and pandemic-related contextual factors. Psychological and psychiatric factors were the most frequently reported domain (28 studies), followed by occupational factors (23 studies), sociodemographic and socioeconomic factors (21 studies), social and organizational factors (14 studies), physical health, sleep, and lifestyle-related factors (11 studies), and early-life adversity and pandemic-related contextual factors (7 studies).

**Table 3:** Summary of factors associated with SI, SP, and SA among nurses

Domain	Reported associated factors	Number of studies
Sociodemographic and socioeconomic factors	Age; gender; marital status; education; income; race/ethnicity or nationality; religion; living condition; family or parental status	21
Psychological and psychiatric factors	Depression; anxiety; stress; psychological distress; burnout; PTSD or secondary traumatic stress; loneliness; resilience; mindfulness; self-efficacy; perceived burdensomeness; thwarted belongingness	28
Physical health, sleep, and lifestyle-related factors	Poor physical health; chronic illness or pain; somatic symptoms; BMI; sleep problems; poor sleep quality; sleep disturbance; short sleep duration; smoking; alcohol, sedative, or substance use	11
Occupational factors	Work department or clinical setting; professional role or title; years of experience; shift work or night shifts; long working hours; workload; job-related stress; job satisfaction; intent to leave; medical errors or adverse events	23
Social and organizational factors	Workplace violence; workplace bullying; workplace sexual harassment; social support; family, colleague, or organizational support; workplace mattering; stigma; discrimination; feeling underappreciated	14
Early-life adversity and pandemic-related contextual factors	Adverse childhood experiences; childhood trauma, abuse, or neglect; COVID-19 exposure; family infection; supporting COVID-19-related departments; perceived infection risk; pandemic-related stress	7

Note. BMI, body mass index; COVID-19, coronavirus disease 2019; PTSD, post-traumatic stress disorder; SA, suicide attempts; SI, suicidal ideation; SP, suicide plans. Studies may contribute to more than one domain due to the multidimensional nature of the factors. A total of 30 independent studies were included after accounting for duplicate dataset reporting

## Discussion

### Principal findings

This systematic review and meta-analysis synthesized evidence on SI, SP, and SA among nurses. The available evidence was uneven across the three outcomes. SI was the most frequently reported outcome, with a pooled prevalence of 12.8%. SP was examined in fewer articles but was also quantitatively synthesized, with a pooled prevalence of 5.0%. SA was summarized narratively because nurse-specific estimates were limited and varied substantially in recall period, sample characteristics, and measurement context. Taken together, these findings suggest that SI and SP are not uncommon among nurses, whereas the available evidence on SA remains limited and heterogeneous. These outcomes warrant continued attention in occupational mental health research, workplace prevention, and healthcare workforce support.

This review also summarized factors reported in relation to SI, SP, and/or SA. These factors covered six broad domains, with psychological and psychiatric factors being the most frequently reported, followed by occupational factors. This finding suggests that suicide-related outcomes among nurses may involve both individual psychological distress and broader occupational and contextual conditions.

Compared with previous reviews that focused on broader healthcare worker populations, this review specifically synthesized evidence on SI, SP, and SA among registered, licensed, or otherwise qualified nurses working in healthcare settings. By focusing on this nurse population, this review provides nurse-specific prevalence estimates and a comprehensive summary of factors associated with suicide-related outcomes. However, evidence remains limited for SP and particularly SA, and further research is needed to better understand suicide-related outcomes among nurses across different settings and contexts.

### Interpretation of heterogeneity

The wide 95% PIs for SI and SP further indicate that prevalence estimates may vary substantially across different nurse populations and study settings. Therefore, the pooled prevalence estimates should be interpreted as summary measures of heterogeneous evidence rather than precise estimates applicable to all contexts. Substantial heterogeneity in SI prevalence across studies appears to arise from multiple levels of variation, including structural (country and regional), methodological (recall period), and study-level factors.

A notable finding of this review is the presence of marked cross-national and regional differences in SI prevalence among nurses, which may partly contribute to the substantial heterogeneity observed across studies. Subgroup analysis showed marked variation in SI prevalence across countries and geographical regions, with higher estimates in Asia than in North America and Europe. In addition, country-level analyses indicated variability across individual countries, with higher estimates observed in countries such as China and Ireland, and lower estimates in countries such as the United States and Spain. However, these comparisons should be interpreted with caution given the limited number of studies available for some countries and potential differences in study design, measurement instruments, and sample characteristics.

Importantly, because study location, geographical region, and broader socioeconomic context are closely interrelated, these differences should not be interpreted as being driven by geography or any single contextual factor. Instead, they may reflect broader structural differences in healthcare systems, socioeconomic development, and nursing workforce conditions across settings.

At the system level, variation in healthcare financing, resource allocation, and nursing workforce capacity may shape differences in occupational environments across countries. At the work environment level, nurses in some Asian settings or resource-constrained healthcare systems may face higher workloads, shift-related strain, inadequate staffing, and limited occupational support, which may be associated with elevated occupational stress, burnout, and psychological burden [75–77]. At the sociocultural level, mental illness-related stigma remains prevalent in some regions and may reduce help-seeking behaviours and mental health service utilization among nurses [78, 79].

Taken together, these findings suggest that observed differences in SI prevalence are likely multifactorial rather than attributable to any single country-level determinant. Given the limited number of studies in some subgroups and persistent within-group heterogeneity, these findings should be interpreted cautiously and regarded as exploratory and hypothesis-generating.

Recall period appeared to be one of the more clearly identified methodological sources of heterogeneity in this review. Subgroup analysis showed significant differences in pooled SI prevalence across recall periods, with relatively higher estimates for past-week and past-two-week SI and lower estimates for past-month and past-year SI. This finding suggests that SI estimates based on different assessment timeframes are not fully comparable, and therefore the overall pooled prevalence should be interpreted with caution. However, except for the past-month subgroup, substantial heterogeneity remained within most recall-period subgroups, indicating that recall period only partially explained between-study differences. Differences in SI prevalence across recall periods may also have been influenced by study region, sample source, measurement instrument, pandemic context, and other study design characteristics. Therefore, these findings should be interpreted as exploratory and should not be taken as evidence that recall period alone accounts for differences in SI prevalence.

Previous ecological momentary assessment studies have shown that SI is not entirely static but may fluctuate over short time scales ranging from hours to days [80]. Shorter recall windows may be more sensitive to recent psychological distress and acute risk states, whereas longer recall periods may reflect broader cumulative exposure and are more susceptible to recall bias and measurement-related differences in reporting [81]. Together, these findings and prior literature suggest that SI prevalence estimates obtained using different recall periods may not reflect the same temporal dimension of risk. Future studies of SI among nurses should use more consistent assessment timeframes and clearly distinguish between recent, past-year, and lifetime SI to improve comparability across studies.

Meta-regression showed a significant negative association between mean age and SI prevalence, suggesting that, at the study level, nurse samples with lower mean age tended to report higher SI prevalence estimates. This finding may reflect differences in sample composition, whereby studies with younger average samples may include more early-career nurses who have relatively limited clinical experience, professional adjustment, job control, and coping resources. Previous studies have shown that younger and early-career nurses may face higher levels of occupational stress and burnout, while burnout, depression, and psychological distress are closely related to SI or suicide risk among nurses [13, 82, 83]. Therefore, the higher SI prevalence observed in samples with lower mean age may point to the potential importance of early-career support, mental health screening, and workplace interventions for younger nurses.

However, this finding should be interpreted cautiously. The meta-regression was based on aggregate study-level data and therefore cannot establish a causal relationship between individual nurses’ age and SI. Nor can it exclude the potential influence of unmeasured factors such as seniority, job position, years of work experience, shift patterns, and sample source. In addition, although mean age explained part of the between-study heterogeneity, residual heterogeneity remained high, indicating that differences in SI prevalence among nurses were still influenced by other study-level, occupational, contextual, and methodological factors.

### Associated factors and implications

The narrative synthesis of associated factors further suggests that suicide-related outcomes among nurses have multilevel characteristics. Although psychological and psychiatric factors were the most frequently reported domain, the extracted factors also involved occupational, social and organizational, physical health and sleep-related, socioeconomic, early-life, and pandemic-related contextual factors (Table 3; Supplementary Table S5). This pattern indicates that SI, SP, and SA among nurses should not be understood solely as manifestations of individual psychological vulnerability, but should be considered in relation to broader occupational, organizational, social, and contextual conditions.

In particular, psychological distress, burnout, sleep problems, and suicide-related outcomes may be shaped by work and organizational environments, including high workload, shift work, long working hours, workplace violence, bullying, and insufficient social or institutional support. Previous studies have also shown that nurses’ psychosocial work environment, occupational burnout, and work stress are associated with SI, and that long working hours and shift work are related to increased SI risk [40, 84]. A recent study among nurses further suggested that burnout, anxiety, depression, post-traumatic stress, and workplace characteristics were associated with SI, whereas resilience, job satisfaction, and workplace mattering may have protective effects [71]. Therefore, prevention strategies should not rely only on individual-level screening or counselling, but should also address modifiable workplace and organizational conditions.

At the individual level, routine and periodic (e.g., annual or post-critical incident) confidential mental health screening may help identify nurses experiencing depression, burnout, sleep problems, psychological distress, or SI at an early stage, particularly among early-career nurses or those exposed to high workload and limited workplace support. Accessible pathways such as occupational health clinics, employee assistance programs, and digital/telehealth counselling services should be made available to facilitate timely support. Relevant professional statements also recommend regular mental health screening through confidential occupational health services and the provision of accessible on-site or virtual mental health services in the workplace [85].

At the organizational level, prevention strategies should address modifiable workplace factors, including excessive workload, frequent night shifts, long working hours, inadequate staffing, workplace violence, bullying, and insufficient supervisor or peer support. Practical measures such as staffing ratio optimization, duty-hour limits, structured rest breaks, anti-bullying policies, and supervisor training in mental health awareness may help reduce occupational stressors. Expert consensus and professional guidance on nurse suicide prevention emphasize the importance of reducing workplace stressors, lowering help-seeking stigma, and strengthening organizational support for nurses at the organizational and system levels [85, 86].

Because stigma, concerns about professional consequences, and limited support resources may prevent nurses from disclosing psychological distress or seeking help, healthcare institutions should provide accessible and confidential support pathways that help reduce stigma [78, 79, 85]. Given the substantial heterogeneity across countries, regions, and study designs, prevention programs should also be adapted to local healthcare systems, workforce resources, workplace cultures, and sociocultural contexts, rather than assuming that a single strategy will be equally applicable to all nurse populations. However, these findings should be interpreted as observed associations rather than causal relationships, given the predominantly cross-sectional nature of the included studies, and should be considered exploratory.

### Strengths and limitations

This review contributes to the evidence on the prevalence of SI, SP, and SA among nurses and summarizes factors associated with these outcomes. By distinguishing SI, SP, and SA in outcome measurement, this review provides a more detailed understanding of different forms of suicide-related risk among nurses. A comprehensive search strategy was applied across multiple databases, including both English- and Chinese-language literature, which enhanced the breadth and representativeness of the included evidence. Random-effects meta-analyses were conducted for SI and SP, and subgroup analyses and meta-regression were further performed to explore potential sources of heterogeneity. These methodological approaches improved the transparency of the analytical process and the interpretability of the findings, and may inform future research and prevention strategies in nursing populations.

However, several limitations should be considered. Substantial heterogeneity was observed across studies, particularly for SI, which may be related to differences in study context, population characteristics, measurement instruments, recall periods, and other methodological factors; therefore, pooled estimates should be interpreted with caution. Most included studies used cross-sectional designs, limiting causal inference regarding factors associated with SI, SP, and SA. In addition, self-reported measures may be subject to recall bias, social desirability bias, and stigma-related underreporting. Variations in recall periods and measurement instruments across studies may also have affected comparability of prevalence estimates. Furthermore, the transformation of multi-category or severity-based outcomes into binary variables may have introduced additional measurement variability. Subgroup analyses by clinical department were not feasible due to insufficient reporting or mixed sampling in many studies. Finally, SP and SA were less frequently studied than SI, limiting the strength of evidence for these outcomes, particularly for SA, which could not be meta-analysed. A proportion of the included studies were conducted during the COVID-19 pandemic, which may have influenced both prevalence estimates and associated factors due to atypical working conditions.

## Conclusions

In summary, this systematic review and meta-analysis found that SI and SP were not uncommon among nurses, with pooled prevalence estimates of 12.8% and 5.0%, respectively. SA was less frequently reported and was summarized narratively due to limited and heterogeneous data. Factors associated with SI, SP, and/or SA covered multiple domains, including psychological and psychiatric, occupational, social and organizational, physical health and sleep-related, sociodemographic, early-life, and pandemic-related contextual factors. These findings suggest that suicide-related outcomes among nurses should be understood as multilevel phenomena, and that prevention strategies should not rely solely on individual-level psychological support, but should also address modifiable workplace and organizational conditions, including work stress, burnout, sleep problems, workplace violence, bullying, and social support. Importantly, these associated factors may also be useful for early identification of high-risk nurses, supporting risk screening and targeted preventive interventions in occupational settings. However, given the substantial heterogeneity across studies and the predominance of cross-sectional evidence, these findings should be interpreted as observed associations rather than causal relationships, and further longitudinal studies are needed to clarify temporal and causal relationships between these factors and SI, SP, and SA among nurses.

## Supplementary Information

Below is the link to the electronic supplementary material.

Supplementary Material 1

## Data Availability

All data generated or analysed during this study are included in this published article and its supplementary information files.

## Abbreviations

CBM: Chinese biomedical literature database
CI: Confidence interval
CNKI: China national knowledge infrastructure
COVID-19: Coronavirus disease 2019
C-SSRS: Columbia-suicide severity rating scale
ICU: Intensive care unit
JBI: Joanna briggs institute
MeSH: Medical subject headings
NA: Not applicable
NOS: Newcastle–ottawa scale
NR: Not reported
PHQ-9: Patient health questionnaire-9
PI: Prediction interval
PRISMA: Preferred reporting items for systematic reviews and meta-analyses
PTSD: Post-traumatic stress disorder
SA: Suicide attempts
SI: Suicidal ideation
SP: Suicide plans
VIP: VIP Chinese Science and Technology Journal Database

## References

[1] World Health Organization. Suicide worldwide in 2021: global health estimates [Internet]. Geneva: World Health Organization; 2025 [cited 2026 Jul 16]. Available from: https://www.who.int/publications/i/item/9789240110069. Accessed 17 Jun 2026.

[2] Nock MK, Borges G, Bromet EJ, Alonso J, Angermeyer M, Beautrais A, et al. Cross-national prevalence and risk factors for suicidal ideation, plans and attempts. Br J Psychiatry. 2008;192(2):98–105. 10.1192/bjp.bp.107.040113. PMC2259024 18245022

[3] Sveticic J, De Leo D. The hypothesis of a continuum in suicidality: a discussion on its validity and practical implications. Ment Illn. 2012;4(2):73–8. 10.4081/mi.2012.e15. PMC4253372 25478116

[4] Peterson C, Sussell A, Li J, Schumacher PK, Yeoman K, Stone DM. Suicide rates by industry and occupation—National Violent Death Reporting System, 32 states, 2016. MMWR Morb Mortal Wkly Rep. 2020;69(3):57–62. 10.15585/mmwr.mm6903a1. PMC7367035 31971929

[5] Boniol M, Kunjumen T, Nair TS, Siyam A, Campbell J, Diallo K. The global health workforce stock and distribution in 2020 and 2030: a threat to equity and ‘universal’ health coverage? BMJ Glob Health. 2022;7(6):e009316. 10.1136/bmjgh-2022-009316. PMC9237893 35760437

[6] Garba S. Our nurses, our future: caring for nurses strengthens economies. Bayero J Nurs Health Care. 2025;6(2):3–5. 10.4314/bjnhc.v6i2.1.

[7] Feng HB, Zhang M, Li XT, Shen Y, Li XH. The level and outcomes of emotional labor in nurses: a scoping review. J Nurs Manag. 2024;2024(1):5317359. 10.1155/2024/5317359. PMC11919068 40224865

[8] Roche N, Darzins S, Oakman J, Stuckey R. Worker experiences of the work health and safety impacts of exposure to dying and death in clinical settings: a qualitative scoping review. Omega J Death Dying. 2025;90(3):1193–228. 10.1177/00302228221117902. 36476137

[9] Berger S, Grzonka P, Frei AI, Hunziker S, Baumann SM, Amacher SA, et al. Violence against healthcare professionals in intensive care units: a systematic review and meta-analysis of frequency, risk factors, interventions, and preventive measures. Crit Care. 2024;28(1):61. 10.1186/s13054-024-04844-z. PMC10898135 38409034

[10] Alimoradi Z, Jafari E, Lin CY, Rajabi R, Marznaki ZH, Soodmand M, et al. Estimation of moral distress among nurses: a systematic review and meta-analysis. Nurs Ethics. 2023;30(3):334–57. 10.1177/09697330221135212. PMC9902807 36704986

[11] Davis MA, Cher BA, Friese CR, Bynum JP. Association of US nurse and physician occupation with risk of suicide. JAMA Psychiatry. 2021;78(6):651–8. 10.1001/jamapsychiatry.2021.0154. PMC8047773 33851982

[12] Davidson JE, Proudfoot J, Lee K, Terterian G, Zisook S. A longitudinal analysis of nurse suicide in the United States (2005–2016) with recommendations for action. Worldviews Evid Based Nurs. 2020;17(1):6–15. 10.1111/wvn.12419. 32017434

[13] Kelsey EA, West CP, Cipriano PF, Peterson C, Satele D, Shanafelt T, et al. Original research: suicidal ideation and attitudes toward help seeking in US nurses relative to the general working population. Am J Nurs. 2021;121(11):24–36. 10.1097/01.NAJ.0000798056.73563.fa. 34629376

[14] Stelnicki AM, Jamshidi L, Angehrn A, Carleton RN. Suicidal behaviors among nurses in Canada. Can J Nurs Res. 2020;52(3):226–36. 10.1177/0844562120934237. 32552154

[15] Brady C, Shackleton E, Fenton C, Loughran O, Hayes B, Hennessy M, et al. Worsening of mental health outcomes in nursing home staff during the COVID-19 pandemic in Ireland. PLoS ONE. 2023;18(9):e0291988. 10.1371/journal.pone.0291988. PMC10521981 37751434

[16] Lu YE, Sun M, Li Y, Wu LL, Zhang X, Wang J, et al. Association of workplace bullying with suicide ideation and attempt among Chinese nurses during the COVID-19 pandemic. J Clin Psychol Med Settings. 2023;30(3):687–96. 10.1007/s10880-022-09915-3. PMC9589744 36272037

[17] Jing S, Dai ZW, Liu XY, Liu X, Zhang L, Wu YJ, et al. Prevalence and influencing factors of suicidal ideation among doctors and nurses during the surge period of the COVID-19 pandemic in China: a multicenter cross-sectional study. QJM. 2025;118(2):97–108. 10.1093/qjmed/hcae190. 39363497

[18] Cheung T, Lee PH, Yip PSF. Suicidality among Hong Kong nurses: prevalence and correlates. J Adv Nurs. 2016;72(4):836–48. 10.1111/jan.12869. 26711369

[19] Alyahya K, Alrefaei R, Almadhyani L, Alquwayz S, Alomairini M, Alsayed F, et al. The prevalence and correlation of suicidal ideation among nurses in King Saud University Medical City. Cureus. 2023;15(9):e44859. 10.7759/cureus.44859. PMC10560092 37809273

[20] Wang J, Zhang X, Yang B, Li JH, Li YY, Chen QY, et al. Suicidal ideation among nurses: unique and cumulative effects of different subtypes of sleep problems. J Affect Disord. 2020;276:600–7. 10.1016/j.jad.2020.07.095. 32871691

[21] Groves S, Lascelles K, Hawton K. Suicide, self-harm, and suicide ideation in nurses and midwives: a systematic review of prevalence, contributory factors, and interventions. J Affect Disord. 2023;331:393–404. 10.1016/j.jad.2023.03.027. 36933670

[22] Basu N, Barinas J, Williams K, Clanton C, Smith PN. Understanding nurse suicide using an ideation-to-action framework: an integrative review. J Adv Nurs. 2023;79(12):4472–88. 10.1111/jan.15681. 37278387

[23] García-Iglesias JJ, Gómez-Salgado J, Fernández-Carrasco FJ, Rodríguez-Díaz L, Vázquez-Lara JM, Prieto-Callejero B, et al. Suicidal ideation and suicide attempts in healthcare professionals during the COVID-19 pandemic: a systematic review. Front Public Health. 2022;10:1043216. 10.3389/fpubh.2022.1043216. PMC9767440 36561871

[24] Kreuze E. Suicidal ideation, suicide planning, and suicide attempt among nurses. Nurs Outlook. 2026;74(1):102616. 10.1016/j.outlook.2025.102616. 41319561

[25] Page MJ, McKenzie JE, Bossuyt PM, Boutron I, Hoffmann TC, Mulrow CD, et al. The PRISMA 2020 statement: an updated guideline for reporting systematic reviews. BMJ. 2021;372:n71. 10.1136/bmj.n71. PMC8005924 33782057

[26] Joanna Briggs Institute. The Joanna Briggs Institute critical appraisal tools for use in JBI systematic reviews: checklist for prevalence studies [Internet]. Adelaide: Joanna Briggs Institute; 2017 [cited 2026 Jul 16]. Available from: https://jbi.global/sites/default/files/2019-05/JBI_Critical_Appraisal-Checklist_for_Prevalence_Studies2017_0.pdf. Accessed 17 Jun 2026.

[27] Wells GA, Shea B, O'Connell D, Peterson J, Welch V, Losos M, et al. The Newcastle-Ottawa Scale (NOS) for assessing the quality of nonrandomised studies in meta-analyses [Internet]. Ottawa: Ottawa Hospital Research Institute; 2000 [cited 2026 Jul 16]. Available from: http://www.ohri.ca/programs/clinical_epidemiology/oxford.asp. Accessed 17 Jun 2026.

[28] Sandeep NP. Exploring the relationship between suicidal thoughts, aggression and distress tolerance among tribal women nurses: a study on tribal cultural context. AJRPC. 2024;5(1):103–24. 10.31920/2634-7644/2024/v5n1a6.

[29] Gonzalez Mendez MJ, Ma L, Alvarado R, Ramirez J, Xu KP, Xu HF, et al. A multi-center study on the negative psychological impact and associated factors in Chinese healthcare workers 1 year after the COVID-19 initial outbreak. Int J Public Health. 2022;67:1604979. 10.3389/ijph.2022.1604979. PMC9454095 36090824

[30] Chan CC, Faherty C, Rahman N, Murrough JW, Benn EK, Clark U, et al. Suicidal ideation among non-physician hospital system staff: prevalence and workplace correlates. J Affect Disord. 2024;362:638–44. 10.1016/j.jad.2024.07.109. PMC11325446 39029665

[31] Villanueva-Blasco VJ, Lozano-Polo A, Guillamó Mínguez C, Villanueva-Silvestre V, Isorna Folgar M, Gea-Caballero V, et al. The healthcare profession as a determinant of depression, suicidal ideation, and drug use in Spain during the COVID-19 pandemic crisis. J Psychoactive Drugs. 2025 Mar 7:1–11. 10.1080/02791072.2025.2474245. 40055881

[32] Lin W, Wang H, Gong L, Lai GY, Zhao XS, Ding H, et al. Work stress, family stress, and suicide ideation: a cross-sectional survey among working women in Shenzhen, China. J Affect Disord. 2020;277:747–54. 10.1016/j.jad.2020.08.081. 32919296

[33] Kabir H, Chowdhury S, Roy AK, Chowdhury S, Islam M, Chomon R, et al. Association of workplace bullying and burnout with nurses’ suicidal ideation in Bangladesh. Sci Rep. 2023;13:14641. 10.1038/s41598-023-41594-4. PMC10480219 37669987

[34] Li L, Ma FF, Yang MJ, Liu XL, Gong CY, Chu JY. Analysis of suicide ideation and its influencing factors among nursing staff of different grades in different hospitals during their career. Zhonghua Lao Dong Wei Sheng Zhi Ye Bing Za Zhi. 2022;40(8):596–601. 10.3760/cma.j.cn121094-20210520-00259. 36052590

[35] Robles R, Fresán A, Alcocer-Castillejos N, Real-Ramírez J, Morales-Chainé S. Brief screening for distress among healthcare professionals: psychometric properties of the Physician Well-Being Index-Spanish version. Int J Environ Res Public Health. 2022;19(15):9451. 10.3390/ijerph19159451. PMC9368125 35954808

[36] Dyrbye L, Johnson P, Johnson L, Satele D, Shanafelt T. Efficacy of the Well-Being Index to identify distress and well-being in U.S. nurses. Nurs Res. 2018;67(6):447–55. 10.1097/NNR.0000000000000313. 30138124

[37] Wang MQ, Wei Z, Wang YF, Sun L. Mediating role of psychological distress in the associations between medical errors, adverse events, suicidal ideation and plan among operating room nurses in China: a cross-sectional study. BMJ Open. 2023;13(6):e069576. 10.1136/bmjopen-2022-069576. PMC10314585 37399442

[38] Kantorski LP, Oliveira MM, Treichel CAS, Bakolis I, Alves PF, Coimbra VCC, et al. Mental health of nursing professionals during the COVID-19 pandemic: a cross-sectional study. Rev Saude Publica. 2022;56:8. 10.11606/s1518-8787.2022056004122. PMC8910133 35293941

[39] Ding CM, Duan ZZ, Luo WQ, Li LD, Li GZ, Li XH, et al. Pathways linking workplace violence and suicidal ideation/non-suicidal self-injury among nurse staff: the mediating role of loneliness and depressive symptoms. BMC Nurs. 2024;23(1):363. 10.1186/s12912-024-02044-2. PMC11141029 38822294

[40] Chin WS, Chen YC, Ho JJ, Cheng NY, Wu HC, Shiao JSC. Psychological work environment and suicidal ideation among nurses in Taiwan. J Nurs Scholarsh. 2019;51(1):106–13. 10.1111/jnu.12441. 30466180

[41] Liu X, Lu S, Dong B, Fang H. Suicidal ideation and its related factors among nurses of oncology departments of four cities in China. China J Mod Med. 2018;28(8):76–81. 10.3969/j.issn.1005-8982.2018.08.016.

[42] Molina JD, Amigo F, Vilagut G, Mortier P, Muñoz-Ruiperez C, Rodrigo Holgado I, et al. Impact of COVID-19 first wave on the mental health of healthcare workers in a front-line Spanish tertiary hospital: lessons learned. Sci Rep. 2024;14(1):8149. 10.1038/s41598-024-58884-0. PMC11001893 38589491

[43] Brady C, Fenton C, Loughran O, Hayes B, Hennessy M, Higgins A, et al. Nursing home staff mental health during the COVID-19 pandemic in the Republic of Ireland. Int J Geriatr Psychiatry. 2022;37(1):1–10. 10.1002/gps.5648. PMC8646737 34729818

[44] Ariapooran S, Ahadi B, Khezeli M. Depression, anxiety, and suicidal ideation in nurses with and without symptoms of secondary traumatic stress during the COVID-19 outbreak. Arch Psychiatr Nurs. 2022;37:76–81. 10.1016/j.apnu.2021.05.005. PMC8938317 35337442

[45] Ling Y, Hou X, Zhang J. Adverse childhood experiences and suicidal ideation among nurses in China. Front Psychiatry. 2025;16:1639762. 10.3389/fpsyt.2025.1639762. PMC12399515 40901259

[46] Freire FO, Marcon SR, Espinosa MM, Santos HGB, Kogien M, Lima NVP, et al. Factors associated with suicide risk among nurses and physicians: a cross-section study. Rev Bras Enferm. 2020;73(Suppl 1):e20200352. 10.1590/0034-7167-2020-0352. 33084840

[47] Kavaliauskas P, Kazlauskas E, Smailyte G. Psychological distress, suicidality and resilience of Lithuanian nurses. BMC Nurs. 2024;23(1):922. 10.1186/s12912-024-02632-2. PMC11656789 39695636

[48] Xu X, Wang W, Chen J, Ai M, Shi L, Wang L, et al. Suicidal and self-harm ideation among Chinese hospital staff during the COVID-19 pandemic: prevalence and correlates. Psychiatry Res. 2021;296:113654. 10.1016/j.psychres.2020.113654. PMC7836678 33360965

[49] Lin P, Chen L, Lin Y, Wu B. Unique and cumulative effects of adverse childhood experiences on suicidal ideation among nurses. J Shandong First Med Univ Shandong Acad Med Sci. 2024;45(11):647–52. 10.3969/j.issn.2097-0005.2024.11.002.

[50] Sadri Damirchi E, Zakibakhsh Mohammadi N, Basir Amir SM. The role of thwarted belongingness, perceived burdensomeness, self-efficacy and ego strength in predicting suicidal ideation of nurses. Health Emerg Disasters Q. 2019;4(2):85–92. 10.32598/hdq.4.2.85.

[51] Olibamoyo O, Coker O, Adewuya A, Ogunlesi O, Sodipo O. Frequency of suicide attempts and attitudes toward suicidal behaviour among doctors and nurses in Lagos, Nigeria. Afr J Psychiatry. 2020;26:1402. 10.4102/sajpsychiatry.v26i0.1402. PMC7433261 32832124

[52] Mao F, Wan J, Sun Y, Yang B, Wang Y, Cao F. Association between transition patterns of sleep problems and suicidal ideation in Chinese female nurses: a prospective study. J Clin Psychol. 2024;80(2):279–90. 10.1002/jclp.23612. 37847787

[53] Wang M, Yan Y, Yang W, He R, Zhao L, Dou Y, et al. Causal mapping of psychological and occupational risk factors for suicidal ideation in psychiatric nurses using Bayesian networks: a multicenter cross-sectional study. PLoS ONE. 2025;20(9):e0333018. 10.1371/journal.pone.0333018. PMC12453236 40982558

[54] He Z, Lei Q, Hu X, Xiong M, Liu J, Wen J, et al. Moral injury and suicidal ideation among health professionals: the role of mental health. J Affect Disord. 2023;333:58–64. 10.1016/j.jad.2023.04.033. PMC10111858 37080491

[55] Bismark M, Scurrah K, Pascoe A, Willis K, Jain R, Smallwood N. Thoughts of suicide or self-harm among Australian healthcare workers during the COVID-19 pandemic. Aust N Z J Psychiatry. 2022;56(12):1555–65. 10.1177/00048674221075540. PMC9679314 35128948

[56] Brady C, Fenton C, Loughran O, Hayes B, Hennessy M, Higgins A, et al. Dublin hospital workers’ mental health during the peak of Ireland’s COVID-19 pandemic. Ir J Med Sci. 2023;192(3):1293–302. 10.1007/s11845-022-03056-0. PMC9217120 35732873

[57] Dobson H, Malpas CB, Burrell AJ, Gurvich C, Chen L, Kulkarni J, et al. Burnout and psychological distress amongst Australian healthcare workers during the COVID-19 pandemic. Australas Psychiatry. 2021;29(1):26–30. 10.1177/1039856220965045. PMC7554409 33043677

[58] Wojda P. Assessment of suicide risk factors among Polish psychiatric nurses. A pilot study. Pol Nurs. 2023;87(1):11–8. 10.20883/pielpol.2023.1.

[59] Karakaya D, Özparlak A, Önder M. Suicide literacy in nurses: a cross-sectional study. J Clin Nurs. 2023;32(1–2):115–25. 10.1111/jocn.16205. 34985161

[60] Stovall M, Hansen L. Suicide risk, changing jobs, or leaving the nursing profession in the aftermath of a patient safety incident. Worldviews Evid Based Nurs. 2021;18(5):264–72. 10.1111/wvn.12534. 34506066

[61] Václavíková K, Kozáková R. Mobbing and its impact on health of nurses: a pilot study. Pielęgniarstwo XXI wieku. 2021;20(3):155–9. 10.2478/pielxxiw-2021-0022

[62] Sánchez Núñez RA, Ramírez C, Gómez Meza MV. Smoking, depression, and suicide risk in the nursing staff of a third-level hospital. Med Univ. 2014;16(63):60–5. https://www.elsevier.es/en-revista-medicina-universitaria-304-articulo-smoking-depression-suicide-risk-in-X1665579614365251

[63] Çelik Durmuş S, Topçu İ, Yıldırım A. Mobbing behaviors encountered by nurses and their effects on nurses. Int J Caring Sci. 2018;11(2):905–13.

[64] Ortiz-Calvo E, Martínez-Alés G, Mediavilla R, González-Gómez E, Fernández-Jiménez E, Bravo-Ortiz MF, et al. The role of social support and resilience in the mental health impact of the COVID-19 pandemic among healthcare workers in Spain. J Psychiatr Res. 2022;148:181–7. 10.1016/j.jpsychires.2021.12.030. PMC8668396 35124398

[65] Chung CC, Lin MF, Ching YC, Kao CC, Chou YY, Ho PH, et al. Mediating and moderating effects of learned resourcefulness on depressive symptoms and positive ideation in hospital nurses in Taiwan. Res Nurs Health. 2012;35(6):576–88. 10.1002/nur.21505. 22911162

[66] Hendrickson RC, Slevin RA, Hoerster KD, Chang BP, Sano E, McCall CA, et al. The impact of the COVID-19 pandemic on mental health, occupational functioning, and professional retention among health care workers and first responders. J Gen Intern Med. 2022;37(2):397–408. 10.1007/s11606-021-07252-z. PMC8675543 34918181

[67] Peng P, Chen Q, Liang M, Liu Y, Chen S, Wang Y, et al. A network analysis of anxiety and depression symptoms among Chinese nurses in the late stage of the COVID-19 pandemic. Front Public Health. 2022;10:996386. 10.3389/fpubh.2022.996386. PMC9667894 36408014

[68] Greenberg N, Weston D, Hall C, Caulfield T, Williamson V, Fong K. Mental health of staff working in intensive care during COVID-19. Occup Med (Lond). 2021;71(2):62–7. 10.1093/occmed/kqaa220. PMC7928568 33434920

[69] Cheng L, Liu R. Workplace sexual harassment and suicide ideation in female nurses: multiplying effects of social stigma and job insecurity. Aust J Psychol. 2025;77(1):2534618. 10.1080/00049530.2025.2534618. PMC12548075 41141374

[70] Zhang S, Zisook S, Davidson J, Shapiro D, Doran N. Suicidal thoughts and behaviors among health care trainees, staff and faculty at an academic medical center. J Clin Med. 2025;14(2):574. 10.3390/jcm14020574. PMC11765537 39860579

[71] Melnyk BM, Davidson JE, Tucker S, Tan A, Hsieh AP, Cooper A, et al. Burnout, mental health, and workplace characteristics: contributors and protective factors associated with suicidal ideation in high-risk nurses. Worldviews Evid Based Nurs. 2025;22(3):e70042. 10.1111/wvn.70042. PMC12140403 40471927

[72] Li X, Ding C, Li G, Duan Z. Psychosocial characteristics pattern correlated with suicidal ideation and non-suicidal self-injury among nurse staff: a latent profile analysis. BMC Nurs. 2024;23(1):280. 10.1186/s12912-024-01970-5. PMC11046841 38671466

[73] Xu H, Liu L, Li Z, Wang X, Li P, Yan K, et al. Network analysis of burnout, depression, and anxiety with occupational and personal outcomes among clinical nurses in China. Sci Rep. 2025;15(1):37042. 10.1038/s41598-025-20865-2. PMC12549974 41131039

[74] Hong S, Ai M, Xu X, Wang W, Chen J, Zhang Q, et al. Immediate psychological impact on nurses working at 42 government-designated hospitals during COVID-19 outbreak in China: a cross-sectional study. Nurs Outlook. 2021;69(1):6–12. 10.1016/j.outlook.2020.07.007. PMC7368912 32919788

[75] Ohue T, Aryamuang S, Bourdeanu L, Church JN, Hassan H, Kownaklai J, et al. Cross-national comparison of factors related to stressors, burnout and turnover among nurses in developed and developing countries. Nurs Open. 2021;8(5):2439–51. 10.1002/nop2.1002. PMC8363416 34310070

[76] Woo T, Ho R, Tang A, Tam W. Global prevalence of burnout symptoms among nurses: a systematic review and meta-analysis. J Psychiatr Res. 2020;123:9–20. 10.1016/j.jpsychires.2019.12.015. 32007680

[77] Aiken LH, Sloane DM, Bruyneel L, Van den Heede K, Griffiths P, Busse R, et al. Nurse staffing and education and hospital mortality in nine European countries: a retrospective observational study. Lancet. 2014;383(9931):1824–30. 10.1016/S0140-6736(13)62631-8. PMC4035380 24581683

[78] Vaishnav M, Javed A, Gupta S, Kumar V, Vaishnav P, Kumar A, et al. Stigma towards mental illness in Asian nations and low- and middle-income countries, and comparison with high-income countries: a literature review and practice implications. Indian J Psychiatry. 2023;65(10):995–1011. 10.4103/indianjpsychiatry.indianjpsychiatry_667_23. PMC10725213 38108051

[79] Javed A, Lee C, Zakaria H, Buenaventura RD, Cetkovich-Bakmas M, Duailibi K, et al. Reducing the stigma of mental health disorders with a focus on low- and middle-income countries. Asian J Psychiatr. 2021;58:102601. 10.1016/j.ajp.2021.102601. 33611083

[80] Czyz EK, King CA, Al-Dajani N, Zimmermann L, Hong V, Nahum-Shani I. Ecological momentary assessments and passive sensing in the prediction of short-term suicidal ideation in young adults. JAMA Netw Open. 2023;6(8):e2328005. 10.1001/jamanetworkopen.2023.28005. PMC10410485 37552477

[81] Hallford DJ, Rusanov D, Winestone B, Kaplan R, Fuller-Tyszkiewicz M, Melvin G. Disclosure of suicidal ideation and behaviours: a systematic review and meta-analysis of prevalence. Clin Psychol Rev. 2023;101:102272. 10.1016/j.cpr.2023.102272. 37001469

[82] Rudman A, Arborelius L, Dahlgren A, Finnes A, Gustavsson P. Consequences of early career nurse burnout: a prospective long-term follow-up on cognitive functions, depressive symptoms, and insomnia. EClinicalMedicine. 2020;27:100565. 10.1016/j.eclinm.2020.100565. PMC7599295 33150328

[83] Ramírez-Elvira S, Romero-Béjar JL, Suleiman-Martos N, Gómez-Urquiza JL, Monsalve-Reyes C, Cañadas-De la Fuente GA, et al. Prevalence, risk factors and burnout levels in intensive care unit nurses: a systematic review and meta-analysis. Int J Environ Res Public Health. 2021;18(21):11432. 10.3390/ijerph182111432. PMC8583312 34769948

[84] Kim J, Kwon R, Yun H, Lim GY, Woo KS, Kim I. The association between long working hours, shift work, and suicidal ideation: a systematic review and meta-analyses. Scand J Work Environ Health. 2024;50(7):503–18. 10.5271/sjweh.4182. PMC11472300 39140746

[85] Kameg B, Kwasky A, Raphel S, Taylor D, ISPN Policy Committee. Caring for our own: promoting mental health and preventing suicide among nursing professionals. Arch Psychiatr Nurs. 2025;59:151985. 10.1016/j.apnu.2025.151985. 41260842

[86] Schimmels J, Groh C, Neft M, Wocial L, Young C, Davidson JE. American Academy of Nursing Expert Panel consensus statement on leveraging equity in policy to improve recognition and treatment of mental health, substance use disorders, and nurse suicide. Nurs Outlook. 2023;71(3):101970. 10.1016/j.outlook.2023.101970. PMC10129051 37104889

