# Supplementary material for: Prevalence and associated factors of suicidal ideation, suicide plans, and suicide attempts among nurses: a systematic review and meta-analysis
Source: BMC Psychiatry. 2026 Jul 30;26:840. doi: 10.1186/s12888-026-08426-x (PMC13721627; doi:10.1186/s12888-026-08426-x)
Supplement: Supplementary file 1 [file 12888_2026_8426_MOESM1_ESM.docx]

Supplementary material for “**Prevalence and associated factors of suicidal ideation, suicide planning, and suicide attempt among nurses: a systematic review and meta-analysis**”

**Table S1.** Detailed search methods

**Table S2a.** Detailed characteristics of included studies

**Table S2b.** Handling of confirmed or potentially overlapping samples in quantitative synthesis

**Table S3.** Prevalence-related data extracted for SI, SP, and SA

**Table S4a.** JBI quality assessment of cross-sectional studies

**Table S4b.** NOS quality assessment of the longitudinal study

**Table S5.** Study-level extraction of factors examined in relation to SI, SP, and SA among nurses, stratified by significance and adjustment status

**Fig. S1.** Leave-one-out sensitivity analysis of SP prevalence

**Table S1.** Detailed search methods

| Database | Search methods |
| --- | --- |
| PubMed | ((("Nurses"[Mesh]) OR (Nurses[Title/Abstract]) OR (Nurse[Title/Abstract]) OR (Nursing Personnel[Title/Abstract]) OR (Personnel, Nursing[Title/Abstract]) OR (Registered Nurses[Title/Abstract]) OR (Nurse, Registered[Title/Abstract]) OR (Nurses, Registered[Title/Abstract]) OR (Registered Nurse[Title/Abstract]))  AND  (("Suicidal Ideation"[Mesh]) OR (Suicidal Ideation[Title/Abstract]) OR (Ideations, Suicidal[Title/Abstract]) OR (Ideation, Suicidal[Title/Abstract]) OR (Suicidal Ideations[Title/Abstract]) OR (Suicidal tendencies[Title/Abstract]) OR (Suicidality[Title/Abstract]) OR (Suicide thoughts[Title/Abstract]) OR (Suicidal thoughts[Title/Abstract]) OR (Suicidal Plan[Title/Abstract]) OR (Suicidal Plans[Title/Abstract]) OR (Suicidal Planning[Title/Abstract]) OR (Suicidal Intentions[Title/Abstract]) OR (Planned Suicide[Title/Abstract]) OR (Suicidal Intent[Title/Abstract]) OR (Suicide Preparation[Title/Abstract]) OR (Suicidal Attempts[Title/Abstract]) OR (Suicide, Attempted[Title/Abstract]) OR (Suicidal Behavior[Title/Abstract]) OR (Suicide Attempt[Title/Abstract]))  AND  (("Prevalence"[Mesh]) OR (Prevalences[Title/Abstract]) OR (Point Prevalence[Title/Abstract]) OR (Point Prevalences[Title/Abstract]) OR (Prevalence, Point[Title/Abstract]) OR (Period Prevalence[Title/Abstract]) OR (Period Prevalences[Title/Abstract]) OR (Prevalence, Period[Title/Abstract]) OR ("Risk Factors"[Mesh]) OR (Factor, Risk[Title/Abstract]) OR (Risk Factor[Title/Abstract]) OR (Population at Risk[Title/Abstract]) OR (Populations at Risk[Title/Abstract]) OR (Risk Scores[Title/Abstract]) OR (Risk Score[Title/Abstract]) OR (Score, Risk[Title/Abstract]) OR (Risk Factor Scores[Title/Abstract]) OR (Risk Factor Score[Title/Abstract]) OR (Score, Risk Factor[Title/Abstract]) OR (Health Correlates[Title/Abstract]) OR (Correlates, Health[Title/Abstract]) OR (Social Risk Factors[Title/Abstract]) OR (Factor, Social Risk[Title/Abstract]) OR (Factors, Social Risk[Title/Abstract]) OR (Risk Factor, Social[Title/Abstract]) OR (Risk Factors, Social[Title/Abstract]) OR (Social Risk Factor[Title/Abstract]))) |
| Web of science | 1: ((((((TS=(nurse)) OR TS=(Nursing Personnel)) OR TS=(Personnel, Nursing)) OR TS=(Registered Nurses)) OR TS=(Nurse, Registered)) OR TS=(Nurses, Registered)) OR TS=(Registered Nurse)  2: (((((((TS=(suicidal ideation)) OR TS=(Ideations, Suicidal)) OR TS=(Ideation, Suicidal)) OR TS=(Suicidal Ideations)) OR TS=(Suicidal tendencies)) OR TS=(Suicidality)) OR TS=(Suicide thoughts)) OR TS=(Suicidal thoughts)  3: ((((TS=(Attempted Suicide)) OR TS=(Suicide Attempt)) OR TS=(Attempt, Suicide)) OR TS=(Parasuicide)) OR TS=(Parasuicides)  4: #2 OR #3  5: (((((((TS=(Prevalence)) OR TS=(Prevalences)) OR TS=(Point Prevalence)) OR TS=(Point Prevalences)) OR TS=(Prevalence, Point)) OR TS=(Period Prevalence)) OR TS=(Period Prevalences)) OR TS=(Prevalence, Period)  6: ((((((((((((((((((TS=(Risk Factors)) OR TS=(Factor, Risk)) OR TS=(Risk Factor)) OR TS=(Population at Risk)) OR TS=(Populations at Risk)) OR TS=(Risk Scores)) OR TS=(Risk Score)) OR TS=(Score, Risk)) OR TS=(Risk Factor Scores)) OR TS=(Risk Factor Score)) OR TS=(Score, Risk Factor)) OR TS=(Health Correlates)) OR TS=(Correlates, Health)) OR TS=(Social Risk Factors)) OR TS=(Factor, Social Risk)) OR TS=(Factors, Social Risk)) OR TS=(Risk Factor, Social)) OR TS=(Risk Factors, Social)) OR TS=(Social Risk Factor)  7: #5 OR #6  8: #1 AND #4 AND #7 |
| Embase | #1. 'nurse'/exp OR 'nurse'  #2. 'nurse',ti OR 'nursing personnel',ti OR 'personnel, nursing',ti OR 'registered nurses',ti OR 'nurse, registered',ti OR 'nurses, registered',ti OR 'registered nurse',ti  #3. #1 OR #2  #4. 'suicidal ideation',ti OR 'ideations, suicidal',ti OR 'ideation, suicidal',ti OR 'suicidal ideations',ti OR 'suicidal tendencies',ti OR 'suicidality',ti OR 'suicide thoughts',ti OR 'suicidal thoughts',ti  #5. 'suicidal ideation'/exp OR 'suicidal ideation'  #6. 'suicide attempt'/exp OR 'suicide attempt'  #7. 'attempted suicide',ti OR 'suicide attempt',ti OR 'attempt, suicide',ti OR 'parasuicide',ti OR 'parasuicides',ti  #8. #4 OR #5 OR #6 OR #7  #9. 'prevalence'/exp  #10. 'prevalence',ti OR 'prevalences',ti OR 'point prevalence',ti OR 'point prevalences',ti OR 'prevalence, point',ti OR 'period prevalence',ti OR 'period prevalences',ti OR 'prevalence, period',ti  #11. 'risk factor'/exp  #12. 'risk factors',ti OR 'factor, risk',ti OR 'risk factor',ti OR 'population at risk',ti OR 'populations at risk',ti OR 'risk scores',ti OR 'risk score',ti OR 'score, risk',ti OR 'risk factor scores',ti OR 'risk factor score',ti OR 'score, risk factor',ti OR 'health correlates',ti OR 'correlates, health',ti OR 'social risk factors',ti OR 'factor, social risk',ti OR 'factors, social risk',ti OR 'risk factor, social',ti OR 'risk factors, social',ti OR 'social risk factor',ti  #13. #9 OR #10 OR #11 OR #12  #14. #3 AND #8 AND #13 |
| PsycINFO | S#: S1  Query (user-entered): MA (nurse)  Query (expanded/display term): MA (nurse)  S#: S2  Query (user-entered): XB (Nurse) OR XB (Nursing Personnel) OR XB (Personnel, Nursing) OR XB (Registered Nurses) OR XB (Nurse, Registered) OR XB (Nurses, Registered) OR XB (Registered Nurse)  Query (expanded/display term): XB (Nurse) OR XB (Nursing Personnel) OR XB (Personnel, Nursing) OR XB (Registered Nurses) OR XB (Nurse, Registered) OR XB (Nurses, Registered) OR XB (Registered Nurse)  S#: S3  Query (user-entered): XB (Suicidal Ideation) OR XB (Ideations, Suicidal) OR XB (Ideation, Suicidal) OR XB (Suicidal Ideations) OR XB (Suicidal tendencies) OR XB (Suicidality) OR XB (Suicide thoughts) OR XB (Suicidal thoughts)  Query (expanded/display term): XB (Suicidal Ideation) OR XB (Ideations, Suicidal) OR XB (Ideation, Suicidal) OR XB (Suicidal Ideations) OR XB (Suicidal tendencies) OR XB (Suicidality) OR XB (Suicide thoughts) OR XB (Suicidal thoughts)  S#: S4  Query (user-entered): MA (Suicidal Ideation)  Query (expanded/display term): MA (Suicidal Ideation)  S#: S5  Query (user-entered): XB (Attempted Suicide) OR XB (Suicide Attempt) OR XB (Attempt, Suicide) OR XB (Parasuicide) OR XB (Parasuicides)  Query (expanded/display term): XB (Attempted Suicide) OR XB (Suicide Attempt) OR XB (Attempt, Suicide) OR XB (Parasuicide) OR XB (Parasuicides)  S#: S6  Query (user-entered): MA (Prevalence)  Query (expanded/display term): MA (Prevalence)  S#: S7  Query (user-entered): XB (Prevalence) OR XB (Prevalences) OR XB (Point Prevalence) OR XB (Point Prevalences) OR XB (Prevalence, Point) OR XB (Period Prevalence) OR XB (Period Prevalences) OR XB (Prevalence, Period)  Query (expanded/display term): XB (Prevalence) OR XB (Prevalences) OR XB (Point Prevalence) OR XB (Point Prevalences) OR XB (Prevalence, Point) OR XB (Period Prevalence) OR XB (Period Prevalences) OR XB (Prevalence, Period)  S#: S8  Query (user-entered): MA (Risk Factors)  Query (expanded/display term): MA (Risk Factors)  S#: S9  Query (user-entered): XB (Risk Factors) OR XB (Factor, Risk) OR XB (Risk Factor) OR XB (Population at Risk) OR XB (Populations at Risk) OR XB (Risk Scores) OR XB (Risk Score) OR XB (Score, Risk) OR XB (Risk Factor Scores) OR XB (Risk Factor Score) OR XB (Score, Risk Factor) OR XB (Health Correlates) OR XB (Correlates, Health) OR XB (Social Risk Factors) OR XB (Factor, Social Risk) OR XB (Factors, Social Risk) OR XB (Risk Factor, Social) OR XB (Risk Factors, Social) OR XB (Social Risk Factor)  Query (expanded/display term): XB (Risk Factors) OR XB (Factor, Risk) OR XB (Risk Factor) OR XB (Population at Risk) OR XB (Populations at Risk) OR XB (Risk Scores) OR XB (Risk Score) OR XB (Score, Risk) OR XB (Risk Factor Scores) OR XB (Risk Factor Score) OR XB (Score, Risk Factor) OR XB (Health Correlates) OR XB (Correlates, Health) OR XB (Social Risk Factors) OR XB (Factor, Social Risk) OR XB (Factors, Social Risk) OR XB (Risk Factor, Social) OR XB (Risk Factors, Social) OR XB (Social Risk Factor)  S#: S10  Query (user-entered): S1 OR S2  Query (expanded/display term): (MA (nurse)) OR (XB (Nurse) OR XB (Nursing Personnel) OR XB (Personnel, Nursing) OR XB (Registered Nurses) OR XB (Nurse, Registered) OR XB (Nurses, Registered) OR XB (Registered Nurse))  S#: S11  Query (user-entered): S3 OR S4 OR S5  Query (expanded/display term): (XB (Suicidal Ideation) OR XB (Ideations, Suicidal) OR XB (Ideation, Suicidal) OR XB (Suicidal Ideations) OR XB (Suicidal tendencies) OR XB (Suicidality) OR XB (Suicide thoughts) OR XB (Suicidal thoughts)) OR (MA (Suicidal Ideation)) OR (XB (Attempted Suicide) OR XB (Suicide Attempt) OR XB (Attempt, Suicide) OR XB (Parasuicide) OR XB (Parasuicides))  S#: S12  Query (user-entered): S6 OR S7 OR S8 OR S9  Query (expanded/display term): (MA (Prevalence)) OR (XB (Prevalence) OR XB (Prevalences) OR XB (Point Prevalence) OR XB (Point Prevalences) OR XB (Prevalence, Point) OR XB (Period Prevalence) OR XB (Period Prevalences) OR XB (Prevalence, Period)) OR (MA (Risk Factors)) OR (XB (Risk Factors) OR XB (Factor, Risk) OR XB (Risk Factor) OR XB (Population at Risk) OR XB (Populations at Risk) OR XB (Risk Scores) OR XB (Risk Score) OR XB (Score, Risk) OR XB (Risk Factor Scores) OR XB (Risk Factor Score) OR XB (Score, Risk Factor) OR XB (Health Correlates) OR XB (Correlates, Health) OR XB (Social Risk Factors) OR XB (Factor, Social Risk) OR XB (Factors, Social Risk) OR XB (Risk Factor, Social) OR XB (Risk Factors, Social) OR XB (Social Risk Factor))  S#: S13  Query (user-entered): S10 AND S11 AND S12  Query (expanded/display term): ((MA (nurse)) OR (XB (Nurse) OR XB (Nursing Personnel) OR XB (Personnel, Nursing) OR XB (Registered Nurses) OR XB (Nurse, Registered) OR XB (Nurses, Registered) OR XB (Registered Nurse))) AND ((XB (Suicidal Ideation) OR XB (Ideations, Suicidal) OR XB (Ideation, Suicidal) OR XB (Suicidal Ideations) OR XB (Suicidal tendencies) OR XB (Suicidality) OR XB (Suicide thoughts) OR XB (Suicidal thoughts)) OR (MA (Suicidal Ideation)) OR (XB (Attempted Suicide) OR XB (Suicide Attempt) OR XB (Attempt, Suicide) OR XB (Parasuicide) OR XB (Parasuicides))) AND ((MA (Prevalence)) OR (XB (Prevalence) OR XB (Prevalences) OR XB (Point Prevalence) OR XB (Point Prevalences) OR XB (Prevalence, Point) OR XB (Period Prevalence) OR XB (Period Prevalences) OR XB (Prevalence, Period)) OR (MA (Risk Factors)) OR (XB (Risk Factors) OR XB (Factor, Risk) OR XB (Risk Factor) OR XB (Population at Risk) OR XB (Populations at Risk) OR XB (Risk Scores) OR XB (Risk Score) OR XB (Score, Risk) OR XB (Risk Factor Scores) OR XB (Risk Factor Score) OR XB (Score, Risk Factor) OR XB (Health Correlates) OR XB (Correlates, Health) OR XB (Social Risk Factors) OR XB (Factor, Social Risk) OR XB (Factors, Social Risk) OR XB (Risk Factor, Social) OR XB (Risk Factors, Social) OR XB (Social Risk Factor))) |
| CNKI and WanFang | Topic = (nurse OR registered nurse OR nursing personnel OR clinical nursing worker)  AND Topic = (suicidal ideation OR suicide OR suicide plan OR suicide attempt OR suicidal thoughts OR suicidal intention)  AND Topic = (prevalence OR risk factors OR associated factors) |
| CBM | 1) "Nurse" [Expanded]  2) "Nurse" OR "Registered Nurse" OR "Nursing Personnel" OR "Clinical Nursing Worker"  3) (#2) OR (#1)  4) "Suicidal Ideation" [Expanded]  5) "Suicide Attempt" [Expanded]  6) "Suicide Plan" OR "Suicidal Thoughts" OR "Suicidal Intention"  7) "Prevalence" [Expanded]  8) "Risk Factors" [Expanded]  9) "Point Prevalence" OR "Prevalence Rate"  10) (#6) OR (#5) OR (#4)  11) (#9) OR (#8) OR (#7)  12) (#11) AND (#10) AND (#3) |
| Scopus | ( ( TITLE-ABS-KEY ( Nurse ) OR TITLE-ABS-KEY ( Nursing Personnel ) OR TITLE-ABS-KEY ( Personnel , Nursing ) OR TITLE-ABS-KEY ( Registered Nurses ) OR TITLE-ABS-KEY ( Nurse , Registered ) OR TITLE-ABS-KEY ( Nurses , Registered ) OR TITLE-ABS-KEY ( Registered Nurse ) ) ) AND ( ( ( TITLE-ABS-KEY ( Suicidal Ideation ) OR TITLE-ABS-KEY ( Ideations , Suicidal ) OR TITLE-ABS-KEY ( Ideation , Suicidal ) OR TITLE-ABS-KEY ( Suicidal Ideations ) OR TITLE-ABS-KEY ( Suicidal tendencies ) OR TITLE-ABS-KEY ( Suicidality ) OR TITLE-ABS-KEY ( Suicide thoughts ) OR TITLE-ABS-KEY ( Suicidal thoughts ) ) ) OR ( ( TITLE-ABS-KEY ( Attempted Suicide ) OR TITLE-ABS-KEY ( Suicide Attempt ) OR TITLE-ABS-KEY ( Attempt , Suicide ) OR TITLE-ABS-KEY ( Parasuicide ) OR TITLE-ABS-KEY ( Parasuicides ) ) ) ) AND ( ( ( TITLE-ABS-KEY ( Prevalence ) OR TITLE-ABS-KEY ( Prevalences ) OR TITLE-ABS-KEY ( Point Prevalence ) OR TITLE-ABS-KEY ( Point Prevalences ) OR TITLE-ABS-KEY ( Prevalence , Point ) OR TITLE-ABS-KEY ( Period Prevalence ) OR TITLE-ABS-KEY ( Period Prevalences ) OR TITLE-ABS-KEY ( Prevalence , Period ) ) ) OR ( ( TITLE-ABS-KEY ( Risk Factors ) OR TITLE-ABS-KEY ( Factor , Risk ) OR TITLE-ABS-KEY ( Risk Factor ) OR TITLE-ABS-KEY ( Population at Risk ) OR TITLE-ABS-KEY ( Populations at Risk ) OR TITLE-ABS-KEY ( Risk Scores ) OR TITLE-ABS-KEY ( Risk SCORE ) OR TITLE-ABS-KEY ( SCORE , Risk ) OR TITLE-ABS-KEY ( Risk Factor Scores ) OR TITLE-ABS-KEY ( Risk Factor SCORE ) OR TITLE-ABS-KEY ( SCORE , Risk Factor ) OR TITLE-ABS-KEY ( Health Correlates ) OR TITLE-ABS-KEY ( Correlates , Health ) OR TITLE-ABS-KEY ( Factor , Social Risk ) OR TITLE-ABS-KEY ( Social Risk Factors ) OR TITLE-ABS-KEY ( Factors , Social Risk ) OR TITLE-ABS-KEY ( Risk Factor , Social ) OR TITLE-ABS-KEY ( Risk Factors , Social ) OR TITLE-ABS-KEY ( Social Risk Factor ) ) ) ) |
| Cochrane | #1 MeSH descriptor: [Nursing] in all MeSH products  #2 (Nurse),ab,kw OR (Nursing Personnel),ab,kw OR (Personnel, Nursing),ab,kw OR (Registered Nurses),ab,kw OR (Nurse, Registered),ab,kw OR (Nurses, Registered),ab,kw  #3 #1 OR #2  #4 MeSH descriptor: [Suicidal Ideation] explode all trees  #5 (Suicidal Ideation),ab,kw OR (Ideations, Suicidal),ab,kw OR (Ideation, Suicidal),ab,kw OR (Suicidal Ideations),ab,kw OR (Suicidal tendencies),ab,kw OR (Suicidality),ab,kw OR (Suicide thoughts),ab,kw OR (Suicidal thoughts),ab,kw  #6 MeSH descriptor: [Suicide, Attempted] explode all trees  #7 (Attempted Suicide),ab,kw OR (Suicide Attempt),ab,kw OR (Attempt, Suicide),ab,kw OR (Parasuicide),ab,kw OR (Parasuicides),ab,kw  #8 #4 OR #5 OR #6 OR #7  #9 MeSH descriptor: [Prevalence] explode all trees  #10 (Prevalence),ab,kw OR (Prevalences),ab,kw OR (Point Prevalence),ab,kw OR (Point Prevalences),ab,kw OR (Prevalence, Point),ab,kw OR (Period Prevalence),ab,kw OR (Period Prevalences),ab,kw OR (Prevalence, Period),ab,kw  #11 MeSH descriptor: [Risk Factors] explode all trees  #12 (Risk Factors),ab,kw OR (Factor, Risk),ab,kw OR (Risk Factor),ab,kw OR (Population at Risk),ab,kw OR (Populations at Risk),ab,kw OR (Risk Scores),ab,kw OR (Risk Score),ab,kw OR (Score, Risk),ab,kw OR (Risk Factor Scores),ab,kw OR (Risk Factor Score),ab,kw OR (Score, Risk Factor),ab,kw OR (Health Correlates),ab,kw OR (Correlates, Health),ab,kw OR (Social Risk Factors),ab,kw OR (Factor, Social Risk),ab,kw OR (Factors, Social Risk),ab,kw OR (Risk Factor, Social),ab,kw  #13 #9 OR #10 OR #11 OR #12  #14 #3 AND #8 AND #13 |
| VIP | ((Abstract = (Nurse OR Clinical Nursing Worker OR Registered Nurse))  AND  (Any Field = (Suicidal Ideation OR Suicide OR Suicide Attempt OR Suicidal Thoughts OR Suicidal Intention))  AND  (Title/Keywords = (Prevalence OR Prevalence Rate OR Point Prevalence OR Risk Factors OR Associated Factors))) |

**Table S2a.** Detailed characteristics of included studies

| Study | Country | Survey years | Pandemic context | Design | Sampling | Setting/participants | Sample size | Age, mean (SD) | Female, n (%) | Outcomes reported |
| --- | --- | --- | --- | --- | --- | --- | --- | --- | --- | --- |
| Sandeep et al. 2024 | India | NR | NR | CS | Non-random | Tribal women nurses working in government and private hospitals | 300 | NR | 300 (100.0) | SI; Associated factors |
| Alyahya et al. 2023 | Saudi Arabia | 2021 | During COVID-19 | CS | Non-random | Hospital nurses | 419 | NR | 381 (90.9) | SI; associated factors |
| Gonzalez Mendez et al. 2022 | China | 2020-2021 | During COVID-19 | CS | Non-random | Nurse subgroup of HCWs | 573 | NR | 533 (93.0) | SI |
| Chan et al. 2024 | United States | 2022 | During COVID-19 | CS | Random | Nurse subgroup of hospital staff | 325 | NR | NR | SI |
| Villanueva-Blasco et al. 2025 | Spain | 2020 | During COVID-19 | CS | Non-random | Nursing professionals | 181 | NR | NR | SI |
| Lin et al. 2020 | China | 2015 | Pre-COVID-19 | CS | Random | Nurse subgroup of working women | 359 | NR | 359 (100.0) | SI |
| Kabir et al. 2023 | Bangladesh | 2021 | During COVID-19 | CS | Non-random | Registered nurses | 1264 | 28.41 (5.54) | 885 (70.0) | SI; associated factors |
| Li et al. 2022 | China | 2020 | During COVID-19 | CS | Random | Nursing staff in secondary/tertiary hospitals | 1644 | 33.15 (6.61) | 1572 (95.6) | SI; associated factors |
| Lu et al. 2023 | China | 2020 | During COVID-19 | CS | Random | Nurses in tertiary hospitals | 1901 | 31.31 (6.04) | 1831 (96.3) | SI; SA; associated factors |
| Robles et al. 2022 | Mexico | 2020 | During COVID-19 | CS | Non-random | Nurse subgroup of healthcare professionals | 1006 | 41.1 (10.6) | 890 (88.5) | SI; associated factors |
| Dyrbye et al. 2018 | United States | 2016 | Pre-COVID-19 | CS | Random | U.S. nurses | 637 | 52.3 (12.5) | NR (95.0) | SI; associated factors |
| Wang et al. 2023 | China | 2021-2022 | During COVID-19 | CS | Non-random | Operating room nurses | 787 | 33.87 (7.07) | 634 (80.6) | SI; SP; associated factors |
| Kantorski et al. 2022 | Brazil | 2020 | During COVID-19 | CS | Non-random | COVID-19-related healthcare settings, Brazil; registered nurses and nurse technicians | 820 | NR | NR | SI |
| Kelsey et al. 2021 | United States | 2017 | Pre-COVID-19 | CS | Random | U.S. nurses | 7378 | 49.6 (16.3)* | 6776 (92.7) | SI; associated factors |
| Ding et al. 2024 | China | 2022 | During COVID-19 | CS | Non-random | Nurse staff in governmental hospitals | 1774 | 32.00 (7.99) | 1666 (93.9) | SI; associated factors |
| Jing et al. 2025 | China | 2023 | During COVID-19 | CS | Non-random | Nurse subgroup of doctors and nurses | 3112 | NR | NR | SI; associated factors |
| Chin et al. 2019 | Taiwan, China | NR | Pre-COVID-19 | CS | Random | Female hospital nurses | 2734 | 32.7 (7.4) | 2734 (100.0) | SI; associated factors |
| Stelnicki et al. 2020 | Canada | 2019 | Pre-COVID-19 | CS | Non-random | Canadian nurses | 3969 | NR | NR (94.3) | SI; SP; SA; associated factors |
| Cheung et al. 2016 | Hong Kong, China | 2013 | Pre-COVID-19 | CS | Non-random | Qualified nurses | 850 | NR | 745 (87.6) | SI; SP; SA; associated factors |
| Liu et al. 2018 | China | 2014 | Pre-COVID-19 | CS | Non-random | Oncology nurses in tertiary hospitals | 1473 | 30.3(8.2) | 1473 (100.0) | SI; associated factors |
| Molina et al. 2024 | Spain | 2020 | During COVID-19 | CS | Non-random | Nurse subgroup of hospital healthcare workers | 260 | NR | NR | SI |
| Brady et al. 2022a | Ireland | 2020- 2021 | During COVID-19 | CS | Non-random | Nurses in nursing homes | 120 | NR | 106 (88.3) | SI; SP |
| Brady et al. 2023 | Ireland | 2021-2022 | During COVID-19 | CS | Non-random | Nurses in nursing homes | 75 | NR | 69 (92.0) | SI; SP |
| Ariapooran et al. 2022 | Iran | 2020 | During COVID-19 | CS | Non-random | Nurses in hospitals of Malayer city | 315 | 34.69 (8.33) | 195 (61.9) | SI; associated factors |
| Ling et al. 2025 | China | 2024 | Post-COVID-19 | CS | Non-random | Registered nurses in two public hospitals | 395 | 26.2 (4.6)* | 319 (80.8) | SI; associated factors |
| Freire et al. 2020 | Brazil | 2018 | Pre-COVID-19 | CS | Non-random | Nurses in a university hospital | 85 | NR | NR | SA |
| Kavaliauskas et al. 2024 | Lithuania | 2021-2022 | During COVID-19 | CS | Non-random | Licensed nurses across Lithuania | 533 | 43.13(12.32) | 521 (97.7) | SP; SA |
| Xu et al. 2021 | China | 2020 | During COVID-19 | CS | Non-random | Nurse subgroup of hospital staff in 46 hospitals | 6241 | NR | NR | SI |
| Lin et al. 2024 | China | 2020 | During COVID-19 | CS | Non-random | Registered nurses in a tertiary general hospital | 744 | 31.13 (6.39) | 722 (97.0) | SI; associated factors |
| Sadri Damirchi et al. 2019 | Iran | 2017 | Pre-COVID-19 | CS | Non-random | Nurses in hospitals of Ardabil | 120 | 33.69 (NR) | 90 (75.0) | SI; associated factors |
| Olibamoyo et al. 2020 | Nigeria | NR | Pre-COVID-19 | CS | Random | Nurses in a tertiary teaching hospital | 115 | 35.81 (7.77) | 101 (87.8) | SA |
| Mao et al. 2024 | China | 2018-2019 | Pre-COVID-19 | LS | Random | Female nurses in tertiary hospitals | 623 | NR | 623 (100.0) | SI; associated factors |
| Wang et al. 2025 | China | 2023 | Post-COVID-19 | CS | Non-random | Psychiatric nurses in 23 psychiatric hospitals and mental health facilities | 1835 | NR | 1627 (88.7) | SI; associated factors |
| He et al. 2023 | China | 2020–2022 | During COVID-19 | CS | Non-random | Nurse subgroup of health professionals | 3578 | NR | NR | SI |
| Bismark et al. 2022 | Australia | 2020 | During COVID-19 | CS | Non-random | Nurse subgroup of frontline healthcare workers | 3053 | NR | NR | SI |
| Brady et al. 2022b | Ireland | 2021 | During COVID-19 | CS | Non-random | Nurse subgroup of Dublin hospital workers | 166 | NR | 154 (92.8) | SI; SP |
| Dobson et al. 2021 | Australia | 2020 | During COVID-19 | CS | Non-random | Nurse subgroup of hospital healthcare workers | 86 | NR | 74 (86.0) | SI |
| Wojda et al. 2023 | Poland | 2021 | During COVID-19 | CS | Non-random | Psychiatric nurses | 190 | NR | 166 (87.4) | SI; associated factors |
| Karakaya et al. 2023 | Turkey | 2020 | Mixed/transition period | CS | Non-random | Nurses in a hospital in Antalya | 348 | NR | 319 (91.7) | SI; SA |
| Stovall et al. 2021 | United States | 2019 | Pre-COVID-19 | CS | Random | Registered nurses involved in patient safety incidents | 237 | 44.0 (NR) | 220 (92.8) | SI; associated factors |
| Václavíková et al. 2021 | Czech Republic | 2020 | During COVID-19 | CS | Non-random | Nurses in acute care units | 84 | 33.25 (8.87) | 76 (90.5) | SI |
| Sánchez-Núñez et al. 2014 | Mexico | 2012–2013 | Pre-COVID-19 | CS | Random | Nursing staff in a third-level university hospital | 232 | 33.4 (12.4) | 193 (83.2) | SI; associated factors |
| Celik Durmus et al. 2018 | Turkey | NR | Pre-COVID-19 | CS | Non-random | Nurses in training and research hospitals | 658 | 31.06 (6.69) | 582 (88.4) | SI |
| Ortiz-Calvo et al. 2022 | Spain | 2020 | During COVID-19 | CS | Non-random | Nurse subgroup of healthcare workers | 727 | NR | NR | SI |
| Chung et al. 2012 | Taiwan, China | 2008 | Pre-COVID-19 | CS | Non-random | Full-time hospital nurses in two teaching hospitals | 658 | 29.43 (6.71) | 653 (99.2) | SI |
| Hendrickson et al. 2022 | United States | 2020–2021 | During COVID-19 | CS | Non-random | RN/LPN subgroup of healthcare workers and first responders | 188 | 42.9(11) | NR | SI |
| Peng et al. 2022 | China | 2020–2021 | During COVID-19 | CS | Non-random | Nurses across China | 6183 | 30.7 (7.4)* | 6023 (97.4) | SI; associated factors |
| Greenberg et al. 2021 | United Kingdom | 2020 | During COVID-19 | CS | Non-random | Nurses working in ICUs across nine NHS hospitals | 344 | NR | NR | SI |
| Cheng et al. 2025 | China | 2024 | Post-COVID-19 | CS | Random | Female nurses in private hospitals | 312 | NR | 312 (100.0) | SI; associated factors |
| Zhang et al. 2025 | United States | 2009-2024 | Mixed/transition period | CS | Non-random | Nursing staff participating in an academic medical-center suicide-risk screening program | 1314 | NR | 1138 (86.6) | SI; SP; SA |
| Melnyk et al. 2025 | United States | NR | NR | CS | Non-random | Moderate-to-high-risk nurses recruited nationally | 499 | 42.0 (11.3) | 459 (92.0) | SI; associated factors |
| Wang et al. 2020 | China | 2018 | Pre-COVID-19 | CS | Random | Nurses in tertiary hospitals in Shandong Province | 1531 | NR | 1496 (97.7) | SI; associated factors |
| Li et al. 2024 | China | 2022 | During COVID-19 | CS | Non-random | Nursing staff in governmental hospitals in Dehong, Yunnan Province | 1774 | 32 (8) | 1666 (93.9) | SI; associated factors |
| Xu et al. 2025 | China | 2020 | During COVID-19 | CS | Non-random | Clinical nurses working in Chinese hospitals | 1975 | 31.7 (8.9)* | 1905 (96.5) | SI; associated factors |
| Hong et al. 2021 | China | 2020 | During COVID-19 | CS | Non-random | Frontline registered nurses in 42 COVID-19 government-designated hospitals | 4692 | NR | 4548 (96.9) | SI; associated factors |

Notes: CS, cross-sectional study; LS, longitudinal study; SI, suicidal ideation; SP, suicide planning; SA, suicide attempt; HCWs, healthcare workers; RN, registered nurse; LPN, licensed practical nurse; ICU, intensive care unit; NHS, National Health Service; NR, not reported. Sample size refers to the nurse sample or nurse subgroup from which suicidal or self-harm behaviour outcomes were extracted. Age is presented as mean (SD) where available. Female sex is presented as n (%) where reported; “NR (%)” indicates that only the percentage was reported in the original study. COVID-19 context was coded according to the reported survey year or survey period as pre-COVID-19, during COVID-19, post-COVID-19, mixed/transition period, or NR. “Associated factors” denotes studies from which factor-related results were extractable. *Age mean and SD were estimated from the reported median, interquartile range, and sample size.

**Table S2b.** Handling of confirmed or potentially overlapping samples

| **Article group** | **Basis for confirmed or potential overlap** | **Article retained in quantitative synthesis** | **Article not simultaneously included** | **Rationale** |
| --- | --- | --- | --- | --- |
| Ding et al. 2024 and Li et al. 2024 | Both articles were based on the same underlying dataset collected from 18 hospitals in Dehong, Yunnan Province, China, with identical sampling frame, study period (July 2022), sample size (n = 1,965 invited; n = 1,774 valid responses), and response rate (90.3%). | Ding et al. 2024 | Li et al. 2024 | Ding et al. 2024 was retained in the SI prevalence meta-analysis because it provided extractable SI prevalence data. Li et al. 2024 was not simultaneously included in the same outcome-specific meta-analysis to avoid double counting, but was retained for the narrative synthesis of associated factors when relevant. |
| Lu et al. 2023 and Wang et al. 2020 | Both articles were based on the Health Longitudinal Survey of Nurses in Shandong Province, China, and recruited nurses from tertiary hospitals using multistage stratified cluster sampling. Although they appeared to use different waves or analytic samples, the shared cohort source and sampling frame indicated potential sample overlap. | Lu et al. 2023 | Wang et al. 2020 | Lu et al. 2023 was retained because it provided a larger analytic sample and more complete suicide-related outcome data, including both SI and SA. Wang et al. 2020 was not simultaneously included in the same outcome-specific meta-analysis to avoid potential double counting, but was retained for the narrative synthesis of associated factors when relevant. |
| Peng et al. 2022 and Xu et al. 2025 | Both articles were based on online cross-sectional surveys of Chinese nurses recruited through WeChat-based snowball sampling during overlapping periods of the COVID-19 pandemic. Peng et al. 2022 recruited nurses from October 2020 to April 2021, whereas Xu et al. 2025 recruited nurses from October 20 to December 29, 2020. The overlapping survey period, similar recruitment method, and overlapping author team indicated potential sample overlap. | Peng et al. 2022 | Xu et al. 2025 | Peng et al. 2022 was retained because it provided a larger nurse sample for the SI prevalence synthesis. Xu et al. 2025 was not simultaneously included in the same outcome-specific meta-analysis to avoid potential double counting, but was retained for the narrative synthesis of associated factors when relevant. |
| Xu et al. 2021 and Hong et al. 2021 | Both articles were based on online surveys conducted among hospital staff or nurses in Chongqing, China, during the early COVID-19 outbreak. Xu et al. 2021 included hospital staff from 46 hospitals, including nurse participants, and collected data from February 14 to March 2, 2020. Hong et al. 2021 focused on frontline nurses from 42 government-designated hospitals and collected data from February 8 to February 14, 2020. The shared region, similar hospital setting, overlapping/adjacent survey period, similar online data collection method, and overlapping author team indicated potential sample overlap. | Xu et al. 2021 | Hong et al. 2021 | Xu et al. 2021 was retained because it provided a larger nurse-specific sample for SI prevalence synthesis. Hong et al. 2021 was not simultaneously included in the same outcome-specific meta-analysis to avoid potential double counting, but was retained for narrative synthesis when relevant. |

Note: Articles listed as “not simultaneously included” were not excluded from the systematic review. They were not included together with the retained article in the same outcome-specific meta-analysis to avoid double counting of confirmed or potentially overlapping nurse samples. When relevant, these articles were retained for narrative synthesis. SI, suicidal ideation; SA, suicide attempt.

**Table S3.** Prevalence-related data extracted for SI, SP, and SA

| Study | Outcome | Recall period | Measurement instrument/item | Overall event/N (%) | Female event/N (%) | Male event/N (%) | Included in meta-analysis |
| --- | --- | --- | --- | --- | --- | --- | --- |
| Sandeep et al. 2024 | SI | NR | SIS-25 | NR/300 (NR) | NR | NA | No |
| Alyahya et al. 2023 | SI | Past week | SIS-10 | 103/419 (24.58) | 94/381 (24.67) | 9/38 (23.68) | Yes |
| Gonzalez Mendez et al. 2022 | SI | Last month | PHQ-9 item 9 | 57/573 (9.9) | NR | NR | Yes |
| Chan et al. 2024 | SI | Past 2 weeks | PHQ-9 item 9 | 29/325 (8.9) | NR | NR | Yes |
| Villanueva-Blasco et al. 2025 | SI | Past 2 weeks | PHQ-9 item 9 | 8/181 (4.4) | NR | NR | Yes |
| Lin et al. 2020 | SI | Past 2 weeks | PHQ-9 item 9 | 70/359 (19.5) | 70/359 (19.5) | NA | Yes |
| Kabir et al. 2023 | SI | NR | SIDAS-5 | 791/1259 (62.8) | NR | NR | Yes |
| Li et al. 2022 | SI | During career | Single-item SI screening question | 170/1644 (10.34) | 165/1572 (10.50) | 5/72 (6.94) | Yes |
| Lu et al. 2023 | SI | Past year | Single-item SI question | 320/1901 (16.8) | 304/1831 (16.6) | 16/70 (22.9) | Yes |
|  | SA | Past year | Single-item SA question | 206/1901 (10.8) | 195/1831 (10.6) | 11/70 (15.7) | No |
| Robles et al. 2022 | SI | Past month | ICD-11 PHC SI item | 98/1006 (9.7) | NR | NR | Yes |
| Dyrbye et al. 2018 | SI | Past year | Single-item SI question | 18/592 (3.0)ᵃ | NR | NR | Yes |
| Wang et al. 2023 | SI | Lifetime/ever | Single-item SI question | 55/787 (7.0) | NR | NR | Yes |
|  | SP | Lifetime/ever | Single-item SP question | 24/787 (3.0) | NR | NR | Yes |
| Kantorski et al. 2022 | SI | Past 30 days | SRQ-20 item 17 | 60/820 (7.3)ᵃ | NR | NR | Yes |
| Kelsey et al. 2021 | SI | Past year | Single-item SI question | 403/7378 (5.5) | 354/6776 (5.2) | 42/530 (7.9) | Yes |
| Ding et al. 2024 | SI | Past year | Single-item SI question | 165/1774 (9.3) | NR | NR | Yes |
| Jing et al. 2025 | SI | Past 2 weeks | Single-item SI question | 875/3112 (28.05) | NR | NR | Yes |
| Chin et al. 2019 | SI | Past week | BPRS SI item | 500/2734 (18.3)ᵃ | 500/2734 (18.3)ᵃ | NA | Yes |
| Stelnicki et al. 2020 | SI | Past year | Single-item SI question | 418/3969 (10.5) | NR | NR | Yes |
|  | SP | Past year | Single-item SP question | 184/3969 (4.6) | NR | NR | Yes |
|  | SA | Past year | Single-item SA question | 27/3969 (0.7) | NR | NR | No |
|  | SI | Lifetime | Single-item SI question | 1309/3969 (33.0) | NR | NR | No |
|  | SP | Lifetime | Single-item SP question | 676/3969 (17.0) | NR | NR | No |
|  | SA | Lifetime | Single-item SA question | 316/3969 (8.0) | NR | NR | No |
| Cheung et al. 2016 | SI | Past year | Single-item SI question | 127/850 (14.9)ᵃ | 119/745 (16.0)ᵃ | 8/105 (7.6)ᵃ | Yes |
|  | SP | Past year | Single-item SP question | 47/850 (5.5)ᵃ | 43/745 (5.8)ᵃ | 4/105 (3.8)ᵃ | Yes |
|  | SA | Past year | Single-item SA question | 35/850 (4.1)ᵃ | 33/745 (4.4)ᵃ | 2/105 (2.0)ᵃ | No |
| Liu et al. 2018 | SI | Past year | Single-item SI question | 99/1473 (6.7) | 99/1473 (6.7) | NA | Yes |
| Molina et al. 2024 | SI | Past 30 days | Modified self-report C-SSRS selected items | 16/260 (6.3)ᵃ | NR | NR | Yes |
| Brady et al. 2022a | SI | Past 7 days | C-SSRS-derived SI item | 16/120 (13.0)ᵃ | NR | NR | Yes |
|  | SP | Past 7 days | C-SSRS-derived SP item | 8/120 (7.0)ᵃ | NR | NR | Yes |
| Brady et al. 2023 | SI | Past 7 days | C-SSRS-derived SI item | 19/75 (25.0)ᵃ | NR | NR | Yes |
|  | SP | Past 7 days | C-SSRS-derived SP item | 12/75 (16.0)ᵃ | NR | NR | Yes |
| Ariapooran et al. 2022 | SI | NR | BSSI | NR/315 (NR) | NR | NR | No |
| Ling et al. 2025 | SI | NR | SIOSS suicide-factor items | NR/395 (NR) | NR | NR | No |
| Freire et al. 2020 | SA | Lifetime | Self-reported lifetime SA item | 8/85 (9.41)ᵃ | NR | NR | No |
| Kavaliauskas et al. 2024 | SP | Lifetime/ever | SBQ-R item | 27/533 (5.1) | NR | NR | Yes |
|  | SA | Lifetime/ever | SBQ-R item | 8/533 (1.5) | NR | NR | No |
| Xu et al. 2021 | SI | Past 2 weeks | PHQ-9 item 9 | 417/6241 (6.68) | NR | NR | Yes |
| Lin et al. 2024 | SI | Past 2 weeks | PHQ-9 item 9 | 105/744 (14.1) | 100/722 (13.9) | 5/22 (22.7) | Yes |
| Sadri Damirchi et al. 2019 | SI | NR | BSSI | NR/120 (NR) | NR | NR | No |
| Olibamoyo et al. 2020 | SA | Lifetime/ever | Self-reported lifetime SA item | 11/115 (9.6) | NR | NR | No |
| Mao et al. 2024 | SI | Past 2 weeks | PHQ-9 item 9 | 88/623 (14.1) | 88/623 (14.1) | NA | No, prospective cohort |
| Wang et al. 2025 | SI | Past 2 weeks | PHQ-9 item 9 | 208/1835 (11.33) | 179/1627 (11.0) | 29/208 (13.94) | Yes |
| He et al. 2023 | SI | Past 2 weeks | PHQ-9 item 9 | 574/3578 (16.0) | NR | NR | Yes |
| Bismark et al. 2022 | SI | Past 2 weeks | PHQ-9 item 9 | 343/3053 (11.2) | NR | NR | Yes |
| Brady et al. 2022b | SI | Past 7 days | C-SSRS SI item | 18/166 (11.0)ᵃ | NR | NR | Yes |
|  | SP | Past 7 days | C-SSRS SP item | 7/166 (4.0)ᵃ | NR | NR | Yes |
| Dobson et al. 2021 | SI | Past 2 weeks | PHQ-9 item 9 | 7/76 (9.2) | NR | NR | Yes |
| Wojda et al. 2023 | SI | NR | Original single-item SI question | 29/190 (15.26) | 21/166 (12.7)ᵃ | 8/24 (33.3)ᵃ | Yes |
| Karakaya et al. 2023 | SI | NR | Self-reported SI history item | 41/348 (11.8) | NR | NR | Yes |
|  | SA | NR | Self-reported SA history item | 11/348 (3.2) | NR | NR | No |
| Stovall et al. 2021 | SI | Past year | SBQ-R item | 62/217 (28.6) | NR | NR | No，selected event-exposed sample |
| Václavíková et al. 2021 | SI | Recent weeks | GHQ-28 suicide-related items | 4/84 (4.76) | NR | NR | Yes |
| Sánchez-Núñez et al. 2014 | SI | Lifetime/ever | Plutchik Suicide Risk Scale SI item | 28/232 (12.1) | NR | NR | Yes |
| Celik Durmus et al. 2018 | SI | NR | Mobbing Scale suicide-related item | 46/658 (7.0)ᵃ | NR | NR | Yes |
| Ortiz-Calvo et al. 2022 | SI | Past month | C-SSRS death wish item | 40/596 (6.7) | NR | NR | Yes |
| Chung et al. 2012 | SI | Past 2 weeks | BDI-II suicidal ideation item | 117/658 (17.8)ᵃ | NR | NR | Yes |
| Hendrickson et al. 2022 | SI | Past 2 weeks | PHQ-9 item 9 | 21/133 (15.8) | NR | NR | Yes |
| Peng et al. 2022 | SI | Past 2 weeks | PHQ-9 item 9 | 1365/6183 (22.08) | NR | NR | Yes |
| Greenberg et al. 2021 | SI | Past 2 weeks | PHQ-9 item 9 | 65/344 (19)ᵃ | NR | NR | Yes |
| Cheng et al. 2025 | SI | NR | SIDAS | NR/312 (NR) | NR | NA | No |
| Zhang et al. 2025 | SI | Past 2 weeks | HEAR suicidal thoughts item | 142/1314 (10.8) | NR | NR | No, self-selected screening sample |
|  | SP | Past 2 weeks | HEAR suicide plan item | 60/1314(4.57) | NR | NR | No, self-selected screening sample |
|  | SA | Past 2 weeks | HEAR suicide action/attempt item | 31/1314 (2.36) | NR | NR | No, self-selected screening sample |
|  | SA | Lifetime | HEAR lifetime suicide attempt item | 114/1314 (8.76) | NR | NR | No, self-selected screening sample |
| Melnyk et al. 2025 | SI | Past 2 weeks | PHQ-9 item 9 | 74/499 (14.8) | 65/460 (14.1) | 8/31 (25.8) | No, high-risk sample |
| Wang et al. 2020 | SI | Past 2 weeks | PHQ-9 item 9 | 165/1531 (10.8) | 161/1496 (10.8) | 2/23 (8.7) | Noᵇ |
| Li et al. 2024 | SI | Past 12 months | Single-item SI question | NR/1774 (NR) | NR | NR | No |
| Xu et al. 2025 | SI | Past 2 weeks | PHQ-9 item 9 | 523/1975 (26.5) | NR | NR | Noᵇ |
| Hong et al. 2021 | SI | Past 2 weeks | PHQ-9 item 9 | 306/4692 (6.5) | 296/4548 (6.5) | 10/144 (6.9) | No |

Note: SRB, suicide-related behavior; SI, suicidal ideation; SP, suicide plan; SA, suicide attempt; NR, not reported; NA, not applicable; PHQ-9, Patient Health Questionnaire-9; SIS, Suicidal Ideation Scale; SIDAS-5, 5-item Suicidal Ideation Attributes Scale; ICD-11 PHC, International Classification of Diseases 11th Revision Primary Health Care; GHQ-28, 28-item General Health Questionnaire; SRQ-20, 20-item Self-Reporting Questionnaire; BPSS, Brief Psychological Safety Scale; BDI-II, Beck Depression Inventory-II; C-SSRS, Columbia-Suicide Severity Rating Scale; BSSI, Beck Scale for Suicide Ideation; SIOSS, Self-rating Idea of Suicide Scale; SBQ-R, Suicide Behaviors Questionnaire-Revised; HEAR, Healer Education Assessment and Referral.

ᵃ Event number was calculated from the reported prevalence and corresponding sample size.

ᵇ Excluded from the main meta-analysis because of possible sample overlap with another retained report.

**Table S4a.** JBI quality assessment of cross-sectional studies

| Study | Q1 | Q2 | Q3 | Q4 | Q5 | Q6 | Q7 | Q8 | Overall methodological quality |
| --- | --- | --- | --- | --- | --- | --- | --- | --- | --- |
| Sandeep et al. 2024 | Yes | Yes | Yes | Yes | No | No | Yes | Yes | 6/8 (Moderate) |
| Alyahya et al. 2023 | Yes | Yes | Yes | Yes | No | No | Yes | Yes | 6/8 (Moderate) |
| Gonzalez Mendez et al. 2022 | Yes | Yes | Yes | Yes | Yes | Yes | Yes | Yes | 8/8 (High) |
| Melnyk et al. 2025 | Yes | Yes | Yes | Yes | No | No | Yes | Yes | 6/8 (Moderate) |
| Chan et al. 2024 | Yes | Yes | Yes | Yes | Yes | Yes | Yes | Yes | 8/8 (High) |
| Wang et al. 2020 | Yes | Yes | Yes | Yes | Yes | Yes | Yes | Yes | 8/8 (High) |
| Villanueva-Blasco et al. 2025 | Yes | Yes | Yes | Yes | Yes | No | Yes | Yes | 7/8 (High) |
| Lin et al. 2020 | Yes | Yes | Yes | Yes | Yes | Yes | Yes | Yes | 8/8 (High) |
| Kabir et al. 2023 | Yes | Yes | Yes | Yes | Yes | Yes | Yes | Yes | 8/8 (High) |
| Li et al. 2022 | Yes | Yes | Yes | Yes | Yes | Yes | Yes | Yes | 8/8 (High) |
| Lu et al. 2023 | Yes | Yes | Yes | Yes | Yes | Yes | Yes | Yes | 8/8 (High) |
| Robles et al. 2022 | Yes | Yes | Yes | Yes | No | No | Yes | Yes | 6/8 (Moderate) |
| Dyrbye et al. 2018 | Yes | Yes | Yes | Yes | No | No | Yes | Yes | 6/8 (Moderate) |
| Wang et al. 2023 | Yes | Yes | Unclear | Yes | Yes | Yes | Yes | Yes | 7/8 (High) |
| Kantorski et al. 2022 | Yes | Yes | Unclear | Yes | Yes | Yes | Yes | Yes | 7/8 (High) |
| Kelsey et al. 2021 | Yes | Yes | Yes | Yes | Yes | Yes | Yes | Yes | 8/8 (High) |
| Ding et al. 2024 / Li et al. 2024 | Yes | Yes | Yes | Yes | Yes | Yes | Yes | Yes | 8/8 (High) |
| Jing et al. 2025 | Yes | Yes | Yes | Yes | Yes | Yes | Yes | Yes | 8/8 (High) |
| Chin et al. 2019 | Yes | Yes | Yes | Yes | Yes | Yes | Yes | Yes | 8/8 (High) |
| Stelnicki et al. 2020 | Yes | Yes | Yes | Yes | Yes | Yes | Yes | Yes | 8/8 (High) |
| Cheung et al. 2016 | Yes | Yes | Yes | Yes | Yes | Yes | Yes | Yes | 8/8 (High) |
| Liu et al. 2018 | Yes | Yes | Yes | Yes | Yes | Yes | Yes | Yes | 8/8 (High) |
| Molina et al. 2024 | Yes | Yes | Yes | Yes | No | No | Yes | Yes | 6/8 (Moderate) |
| Brady et al. 2022a | Yes | Yes | Yes | Yes | Yes | Yes | Yes | Yes | 8/8 (High) |
| Brady et al. 2023 | Yes | Yes | Yes | Yes | No | No | Yes | Yes | 6/8 (Moderate) |
| Ariapooran et al. 2022 | Yes | Yes | Yes | Yes | Yes | Yes | Yes | Yes | 8/8 (High) |
| Ling et al. 2025 | Yes | Yes | Yes | Yes | No | No | Yes | Yes | 6/8 (Moderate) |
| Freire et al. 2020 | Yes | Yes | Yes | Yes | Yes | Yes | Yes | Yes | 8/8 (High) |
| Kavaliauskas et al. 2024 | Yes | Yes | Unclear | Yes | Yes | Yes | Yes | Yes | 7/8 (High) |
| Xu et al. 2021 | Yes | Yes | Unclear | Yes | Yes | Yes | Yes | Yes | 7/8 (High) |
| Lin et al. 2024 | Yes | Yes | Yes | Yes | Yes | Yes | Yes | Yes | 8/8 (High) |
| Sadri Damirchi et al. 2019 | Yes | Unclear | Yes | Yes | No | No | Yes | Yes | 5/8 (Moderate) |
| Olibamoyo et al. 2020 | Yes | Yes | Unclear | Yes | Yes | Yes | Yes | Yes | 7/8 (High) |
| Xu et al. 2025 | Yes | Yes | Yes | Yes | No | No | Yes | Yes | 6/8 (Moderate) |
| Wang et al. 2025 | Yes | Yes | Yes | Yes | Yes | Yes | Yes | Yes | 8/8 (High) |
| He et al. 2023 | Yes | Yes | Yes | Yes | Yes | Yes | Yes | Yes | 8/8 (High) |
| Bismark et al. 2022 | Yes | Yes | Yes | Yes | Yes | Yes | Yes | Yes | 8/8 (High) |
| Brady et al. 2022b | Yes | Yes | Yes | Yes | Yes | Yes | Yes | Yes | 8/8 (High) |
| Dobson et al. 2021 | Yes | Yes | Yes | Yes | Yes | Yes | Yes | Yes | 8/8 (High) |
| Wojda et al. 2023 | Yes | Yes | Yes | Unclear | Yes | Yes | Unclear | Yes | 6/8 (Moderate) |
| Karakaya et al. 2023 | Yes | Yes | Yes | Unclear | Yes | Yes | Unclear | Yes | 6/8 (Moderate) |
| Stovall et al. 2021 | Yes | Yes | Unclear | Yes | Unclear | Yes | Yes | Yes | 6/8 (Moderate) |
| Václavíková et al. 2021 | Yes | Yes | Yes | Yes | No | No | Yes | Yes | 6/8 (Moderate) |
| Sánchez-Núñez et al. 2014 | Yes | Yes | Yes | Yes | Yes | Yes | Yes | Yes | 8/8 (High) |
| Celik Durmus et al. 2018 | Yes | Yes | Yes | Yes | No | No | Unclear | Yes | 5/8 (Moderate) |
| Ortiz-Calvo et al. 2022 | Yes | Yes | Unclear | Yes | Yes | Yes | Yes | Yes | 7/8 (High) |
| Chung et al. 2012 | Yes | Yes | Yes | Yes | Yes | Yes | Yes | Yes | 8/8 (High) |
| Hendrickson et al. 2022 | Yes | Yes | Unclear | Yes | Yes | Yes | Yes | Yes | 7/8 (High) |
| Peng et al. 2022 | Yes | Yes | Yes | Yes | No | No | Yes | Yes | 6/8 (Moderate) |
| Hong et al. 2021 | Yes | Yes | Unclear | Yes | Yes | Yes | Yes | Yes | 7/8 (High) |
| Greenberg et al. 2021 | Yes | Yes | Yes | Yes | No | No | Yes | Yes | 6/8 (Moderate) |
| Cheng et al. 2025 | Yes | Yes | Yes | Yes | No | No | Yes | Yes | 6/8 (Moderate) |
| Zhang et al. 2025 | Yes | Yes | Yes | Yes | Yes | Yes | Yes | Yes | 8/8 (High) |

Notes: JBI, Joanna Briggs Institute. Q1, Were the criteria for inclusion in the sample clearly defined? Q2, Were the study subjects and the setting described in detail? Q3, Was the exposure measured in a valid and reliable way? Q4, Were objective, standard criteria used for measurement of the condition? Q5, Were confounding factors identified? Q6, Were strategies to deal with confounding factors stated? Q7, Were the outcomes measured in a valid and reliable way? Q8, Was appropriate statistical analysis used? Each item was rated as “Yes”, “No”, “Unclear”, or “Not applicable”. “Yes” was scored as 1, whereas “No” and “Unclear” were scored as 0. The total score was calculated as the number of “Yes” responses across the eight JBI items. Overall methodological quality was classified as high for scores of 7–8, moderate for scores of 4–6, and low for scores of 0–3. Studies with fewer than four “Yes” responses were excluded from the final review.

**Table S4b.** NOS quality assessment of the longitudinal study

| **Study** | **S1** | **S2** | **S3** | **S4** | **C1** | **C2** | **O1** | **O2** | **O3** | **Total NOS score** | **Overall methodological quality** |
| --- | --- | --- | --- | --- | --- | --- | --- | --- | --- | --- | --- |
| Mao et al. 2024 | ★ | ★ | ★ | ★ | ★ | ★ | – | ★ | – | 7/9 | Acceptable |

Notes: NOS, Newcastle–Ottawa Scale. S1, representativeness of the exposed cohort; S2, selection of the non-exposed cohort; S3, ascertainment of exposure; S4, demonstration that the outcome of interest was not present at baseline; C1, comparability of cohorts for the most important factor; C2, comparability of cohorts for additional factors; O1, assessment of outcome; O2, follow-up long enough for outcomes to occur; O3, adequacy of follow-up. ★ indicates that the criterion was met; – indicates that no star was awarded. A total NOS score of ≥6 was considered to indicate acceptable methodological quality.

**Table S5.** Study-level extraction of factors examined in relation to SI, SP, and SA among nurses, stratified by significance and adjustment status

| **Study** | **Significant adjusted associations** | **Significant unadjusted associations** | **Reported non-significant associations** | **Factor domains** |
| --- | --- | --- | --- | --- |
| Sandeep et al. 2024 | Aggression; distress tolerance | Hospital sector/institution type; aggression; distress tolerance | NR | Social and organizational factors; psychological and psychiatric factors |
| Alyahya et al. 2023 | NR | Marital status; religion; living condition; depression; anxiety; stress | Gender; age; nationality; department; years of experience | Sociodemographic and socioeconomic factors; psychological and psychiatric factors; occupational factors |
| Kabir et al. 2023 | Workplace bullying; burnout; workplace bullying × burnout interaction; sex; monthly income; administrative division of workplace; work department; insufficient equipment to manage patients | Workplace bullying; burnout; age; monthly income; type of job; level of hospital; administrative division of workplace; work department; years of experience; insufficient equipment to manage patients | Educational level; marital status; smoking status; weekly working hours | Sociodemographic and socioeconomic factors; psychological and psychiatric factors; occupational factors; social and organizational factors |
| Li et al. 2022 | Marital status; night shift; self-rated social status; physical disease; social support; mental health status | Marital status; nursing level; smoking status; drinking status; physical disease; self-rated social status; self-rated practice environment; social support; mental health status | Gender; administrative position; education level; professional title; monthly income; department; employment status; age; coping style; some factors differed by hospital level | Sociodemographic and socioeconomic factors; psychological and psychiatric factors; physical health, sleep, and lifestyle-related factors; occupational factors; social and organizational factors |
| Lu et al. 2023 | Workplace bullying; individuals’ isolation from work; attack on personality; direct negative behaviors; number of bullying subtypes | Type of nursing role; monthly income; somatic subhealth symptoms; sleep problems; anxiety symptoms; depressive symptoms; one bullying subtype | Age; sex; marital status; BMI; pregnancy; education level; years of nursing practice; number of night shifts; attack on professional status | Sociodemographic and socioeconomic factors; psychological and psychiatric factors; physical health, sleep, and lifestyle-related factors; occupational factors; social and organizational factors |
| Robles et al. 2022 | NR | Psychological distress measured by PWBI-S cutoff scores | NR | Psychological and psychiatric factors |
| Dyrbye et al. 2018 | NR | Higher distress / lower well-being measured by WBI score | NR | Psychological and psychiatric factors |
| Wang et al. 2023 | Psychological distress; medical errors; adverse events | NR | Age; gender; marital status; occupation/managerial role; professional title; work years | Sociodemographic and socioeconomic factors; psychological and psychiatric factors; occupational factors |
| Kelsey et al. 2021 | White race/ethnicity; DNP degree; burnout; positive depression screen | Age; gender; relationship status; having children; age of youngest child; years of nursing experience; APRN certification; mandatory or unplanned overtime; high emotional exhaustion; high depersonalization | Hispanic ethnicity; hours worked per week; primary practice setting | Sociodemographic and socioeconomic factors; psychological and psychiatric factors; occupational factors |
| Ding et al. 2024 | Workplace violence; loneliness; depressive symptoms | NR | NR | Psychological and psychiatric factors; social and organizational factors; occupational factors |
| Jing et al. 2025 | Supporting other departments that treated COVID-19 patients; anxiety symptoms; burnout; resilience; mindfulness; perceived social support | NR | Gender; education; income; hospital department; years of working; treating COVID-19 patients in own department; perceived higher work intensity; COVID-19 infection status; doses of COVID-19 vaccine; PTSD symptoms; age | Sociodemographic and socioeconomic factors; psychological and psychiatric factors; occupational factors; early-life adversity and pandemic-related contextual factors |
| Chin et al. 2019 | Educational level of junior college or below; high personal burnout; high client-related burnout; always feeling stressed at work | Age 36–40 years; working >60 hours in the past week; sleep duration <6 hours per working day; job dissatisfaction | Shift work | Sociodemographic and socioeconomic factors; psychological and psychiatric factors; physical health, sleep, and lifestyle-related factors; occupational factors |
| Stelnicki et al. 2020 | Age; marital status; provincial region; nurse type; years of service; positive screen for PTSD; positive screen for MDD; positive screen for GAD; positive screen for panic disorder; positive screen for alcohol use disorder | NR | Gender; work location | Sociodemographic and socioeconomic factors; psychological and psychiatric factors; occupational factors |
| Cheung et al. 2016 | Female gender; religion; marital status/having a partner; ward manager/APN position; shift work; experience of patients’ self-harm/suicide; marital disturbance; family relationship crisis; bereavement; chronic illness/health disturbance; debt; deliberate self-harm; alcohol consumption; depressive symptoms; anxiety symptoms; poor self-rated physical health; poor self-rated mental health; help-seeking behavior related to DSH/suicide | Job dissatisfaction; disturbance with colleagues; career disturbance; chronic pain/disability; serious injury/disability; family or friends’ deliberate self-harm; hypnotic consumption | Age; monthly household income; academic qualification; clinical experience; workplace violence; smoking | Sociodemographic and socioeconomic factors; psychological and psychiatric factors; physical health, sleep, and lifestyle-related factors; occupational factors; social and organizational factors |
| Liu et al. 2018 | Marital status of remarriage, cohabitation, separation/divorce, or widowhood; attending nurse technical title; depression; CNSS family domain score >62; CNSS patient-condition domain score >123; CNSS workload domain score >104 | Poor family economic condition; years of nursing ≤8 years; frequent night shifts; CNSS interpersonal relationship domain score >85 | Age; educational level; hospital type/sample source; oncology department type; employment type; CNSS work-expectation domain; CNSS work-nature domain | Sociodemographic and socioeconomic factors; psychological and psychiatric factors; occupational factors; social and organizational factors |
| Ariapooran et al. 2022 | Secondary traumatic stress symptoms | NR | NR | Psychological and psychiatric factors; occupational factors; early-life adversity and pandemic-related contextual factors |
| Ling et al. 2025 | Adverse childhood experiences; psychological strain; ACEs × impulsivity interaction | Impulsivity | NR | Psychological and psychiatric factors; early-life adversity and pandemic-related contextual factors |
| Lin et al. 2024 | Childhood emotional abuse; cumulative ACEs / number of ACEs | BMI ≥24 kg/m²; attending nurse technical title; depressive symptoms; lower family monthly income; physical abuse; sexual abuse; emotional neglect; physical neglect; family violence; isolation/rejection; low socioeconomic status | Age; gender; marital status; residence; ethnicity; education level; work years; parental separation/divorce; family substance abuse; family mental illness; family criminality; peer bullying; witnessing community violence | Sociodemographic and socioeconomic factors; psychological and psychiatric factors; occupational factors; early-life adversity and pandemic-related contextual factors |
| Sadri Damirchi et al. 2019 | Thwarted belongingness; perceived burdensomeness; lower self-efficacy | Lower ego strength | NR | Psychological and psychiatric factors; social and organizational factors |
| Mao et al. 2024 | Global sleep problem score; deteriorated global sleep problems; chronic global sleep problems; sleep disturbance; deteriorated sleep disturbance; chronic sleep disturbance; short sleep duration/deteriorated sleep duration; chronic short sleep duration; use of sleep medication; cumulative number of sleep component problems | Years of working; depressive symptoms | Age; ethnicity; marital status; education status; monthly income; frequency of shift work; subjective sleep quality; sleep latency; sleep efficiency; daytime dysfunction; released sleep problems | Sociodemographic and socioeconomic factors; psychological and psychiatric factors; physical health, sleep, and lifestyle-related factors; occupational factors |
| Wang et al. 2025 | Emotional exhaustion; depersonalization; personal accomplishment dimension; stress at work; general well-being; home-work interface; sad mood; low energy; guilt; psychomotor changes; appetite changes | Unmarried or divorced status; lower income; workplace violence; poorer working conditions; lower control at work; lower employee engagement; lower job and career satisfaction | Gender; age; education level; professional title; work experience; night shift frequency; overtime | Sociodemographic and socioeconomic factors; psychological and psychiatric factors; occupational factors; social and organizational factors |
| Wojda et al. 2023 | NR | Male gender; adverse perceived impact of psychiatric work on health; work-related anxiety/fear; diagnosed mental disorder; depression; addiction to psychoactive substances; use of sedatives to cope with stress; alcohol use to cope with stress; lack of workplace support; feeling underappreciated at work | Psychiatry work experience; place of employment; education level; age; marital status; work-life/private-life interference | Sociodemographic and socioeconomic factors; psychological and psychiatric factors; physical health, sleep, and lifestyle-related factors; occupational factors; social and organizational factors |
| Stovall et al. 2021 | Patient safety incident resulting in patient death | Intention to leave the nursing profession after a patient safety incident | Age; years licensed as an RN; highest earned nursing degree; patient safety incident resulting in temporary harm; patient safety incident resulting in permanent harm; unknown degree of patient harm | Sociodemographic and socioeconomic factors; occupational factors; social and organizational factors |
| Sánchez-Núñez et al. 2014 | Younger age; depressive symptoms / feeling down and sad | Active smoking / nicotine dependence; operational depression | NR | Sociodemographic and socioeconomic factors; psychological and psychiatric factors; physical health, sleep, and lifestyle-related factors |
| Peng et al. 2022 | Restlessness; nervousness; feeling afraid; uncontrollable worry | NR | NR | Psychological and psychiatric factors; early-life adversity and pandemic-related contextual factors |
| Cheng et al. 2025 | Workplace sexual harassment; sexual harassment by colleagues and patients; social stigma; job insecurity; workplace sexual harassment × job insecurity interaction | NR | NR | Psychological and psychiatric factors; occupational factors; social and organizational factors |
| Melnyk et al. 2025 | NR | Never married status; staff nurse role; licensed nurse role; 12-h or longer shifts; burnout; anxiety symptoms; depressive symptoms; post-traumatic stress symptoms; intent to leave job; lower job satisfaction; lower workplace mattering; lower resilience; fewer healthy lifestyle behaviors; lower health-related personal beliefs | Age; gender; race/ethnicity; separated/divorced/widowed marital status; advanced practice nurse role; other nurse role | Sociodemographic and socioeconomic factors; psychological and psychiatric factors; physical health, sleep, and lifestyle-related factors; occupational factors; social and organizational factors |
| Wang et al. 2020 | Overall sleep problems / higher PSQI global score; cannot breathe comfortably at night; cumulative number of sleep problem subtypes | Age 26–45 years; married or living with a partner; having an oldest child >5 years; longer current work tenure; intermediate professional status; depressive symptoms; bad dreams | Gender; education background; employment status; position title; number of night shifts per month; most individual sleep-problem subtypes after full adjustment except cannot breathe comfortably; one subtype of sleep problems | Sociodemographic and socioeconomic factors; psychological and psychiatric factors; physical health, sleep, and lifestyle-related factors; occupational factors |
| Li et al. 2024 | Emotional problem profile; childhood trauma profile; resilience and social support profile; divorced/other marital status; unmarried status | NR | Age; sex; ethnicity; residence; education level; only-child status; monthly income | Sociodemographic and socioeconomic factors; psychological and psychiatric factors; physical health, sleep, and lifestyle-related factors; social and organizational factors; early-life adversity and pandemic-related contextual factors |
| Xu et al. 2025 | Worthlessness; feeling afraid; restlessness; emotional exhaustion; depersonalization | NR | NR | Psychological and psychiatric factors; occupational factors |
| Hong et al. 2021 | Poor/fair subjective health status; family member infected with COVID-19; insufficient family support; insufficient hospital authority support; insufficient support/opportunities for expressing opinions through mass media; high job-related stress | High perceived possibility of infection; encountered discrimination; insufficient support from colleagues; insufficient support from patients; insufficient support from insurance and compensation | Gender; marital status; educational level | Sociodemographic and socioeconomic factors; physical health, sleep, and lifestyle-related factors; occupational factors; social and organizational factors; early-life adversity and pandemic-related contextual factors |

Note: SI, suicidal ideation; SP, suicide plan; SA, suicide attempt; NR, not reported; ACEs, adverse childhood experiences; APN, advanced practice nurse; APRN, advanced practice registered nurse; BMI, body mass index; CNSS, Chinese Nurses Stressor Scale; COVID-19, coronavirus disease 2019; DNP, Doctor of Nursing Practice; DSH, deliberate self-harm; GAD, generalized anxiety disorder; MDD, major depressive disorder; PSQI, Pittsburgh Sleep Quality Index; PTSD, post-traumatic stress disorder; PWBI-S, Physician Well-Being Index score; RN, registered nurse; WBI, Well-Being Index. Studies may contribute to more than one factor domain because associated factors were multidimensional. Factors are listed according to the statistical results reported in each original study, and no causal interpretation should be inferred.

**Fig. S1.** Leave-one-out sensitivity analysis of SP prevalence
